# A bioinspired flexible neuromuscular system based thermal-annealing-free perovskite with passivation

**DOI:** 10.1038/s41467-022-35092-w

**Published:** 2022-12-02

**Authors:** Jiaqi Liu, Jiangdong Gong, Huanhuan Wei, Yameng Li, Haixia Wu, Chengpeng Jiang, Yuelong Li, Wentao Xu

**Affiliations:** 1 Institute of Photoelectronic Thin Film Devices and Technology of Nankai University; Solar Energy Research Center of Nankai University; Key Laboratory of Photoelectronic Thin Film Devices and Technology of Tianjin; Engineering Research Center of Thin Film Photoelectronic Technology, Ministry of Education, #38 Tongyan Road, Jinnan District, Tianjin, 300350 China; 2 Shenzhen Research Institute of Nankai University, Shenzhen, 518000 China; 3 Smart Sensing Interdisciplinary Science Center, Nankai University, Tianjin, 300350 China; 4 College of Chemical and Pharmaceutical Engineering, Hebei University of Science and Technology, Shijiazhuang, 050018 Hebei China

**Keywords:** Organic-inorganic nanostructures, Electronic devices

## Abstract

Brain-inspired electronics require artificial synapses that have ultra-low energy consumption, high operating speed, and stable flexibility. Here, we demonstrate a flexible artificial synapse that uses a rapidly crystallized perovskite layer at room temperature. The device achieves a series of synaptic functions, including logical operations, temporal and spatial rules, and associative learning. Passivation using phenethyl-ammonium iodide eliminated defects and charge traps to reduce the energy consumption to 13.5 aJ per synaptic event, which is the world record for two-terminal artificial synapses. At this ultralow energy consumption, the device achieves ultrafast response frequency of up to 4.17 MHz; which is orders of magnitude magnitudes higher than previous perovskite artificial synapses. A multi-stimulus accumulative artificial neuromuscular system was then fabricated using the perovskite synapse as a key processing unit to control electrochemical artificial muscles, and realized muscular-fatigue warning. This artificial synapse will have applications in future bio-inspired electronics and neurorobots.

## Abstract (web summary)

Designing efficient brain-inspired electronics remains a challenge. Here, Liu et al. develop a flexible perovskite-based artificial synapse with low energy consumption and fast response frequency and realize an artificial neuromuscular system with muscular-fatigue warning.

## Introduction

A human brain uses only 1/10^5^ as much energy to solve the same problem as does a Von Neumann computer^1–3^, which relies on complex algorithms and serial processing^4,5^. Therefore, brain-inspired electronics has become a promising Post-Moorish solution^6–8^, in which the energy consumption of information processing and memory units, i.e., artificial synapses, is an important concern to determine the system-level energy efficiency^9–13^.

Organic–inorganic hybrid perovskites (OHPs) are promising materials to meet this need. These characteristics of low activation energy of X and mixed ionic/charge carrier conductivity^14,15^, make OHPs a good candidate for low-energy-consumption artificial synapses^16,17^. However, due to low defect-formation energy, perovskite films fabricated using the traditional solution process include tremendous defects, which act as non-radiative recombination centres^18^, accelerate the degradation of perovskite and prevent their use in practical situations^19^. Meanwhile, the expenditure of synaptic energy is also increased if too many ions hop between defects. Therefore, if the formation of these intrinsic defects can be averted, the energy consumption could be reduced and the stability of PVK synaptic devices can be improved.

Although OHPs can be easily synthesized using solution-processing techniques, traditional perovskite precursor inks usually use solvents that have high boiling temperature, so the fabrication process requires solvent-removal steps at elevated temperatures and relatively long processing time to promote crystal growth thermodynamically^15,20–22^. The preparation of such perovskites usually requires multiple steps, and therefore does not favor use of perovskites in flexible electronic devices^17,23^, on plastic substrates^24^ that have low glass transition temperatures *T*_g_. These problems indicate the necessity of passivating the defects to reduce energy consumption, and achieve a process to prepare high-quality perovskite semiconductor films that can proceed spontaneously at room temperature to fabricate flexible electronic devices on low-*T*_g_ plastic substrates.

Moreover, next-generation bioinspired robots require physical intelligence (PI) in addition to the computational intelligence (CI) in brain^25,26^. Some preliminary attempts have been made to construct artificial neuromuscular systems, but most of them simply implement unconditioned movements, without higher-level motion-status feedback logic. This movement mode without PI cannot accurately judge the motion status and the deflection limit of the actuator, and therefore reduces the operational safety of bioinspired robots. When human muscle fiber cells are tensioned at high intensity or for a long time, the nervous system increases its secretion of adenosine, which causes perception of fatigue, and encourages the user to relax muscle fibers and avoid overwork injury^27,28^. This kind of fatigue-warning ability is desired for the next generation of flexible bioinspired robots and biological system^29^.

In this work, we demonstrate a flexible perovskite artificial synapse that uses a room-temperature (RT) fabrication process. Benefiting from the transformative manufacturing strategy, the OHPs film can crystallize rapidly within 10 s at RT without any heating process. A phenethyl ammonium iodide (PEAI) layer on [00*l*]-orientation-dominated OHP thin film passivates defects and reduce the energy consumption of a single synaptic event down to so-far the lowest value ~13.5 aJ. The PEAI passivation layer also increased the flexibility of the device, which showed negligible decay after 2000 bending cycles. A multi-stimulus accumulative artificial neuromuscular system was then fabricated using the artificial synapses served as key information processing units to control the actions of electrochemical artificial muscles. The artificial neuromuscular system showed special muscular-fatigue warning ability.

## Results

### General concept

**Fig. 1:**
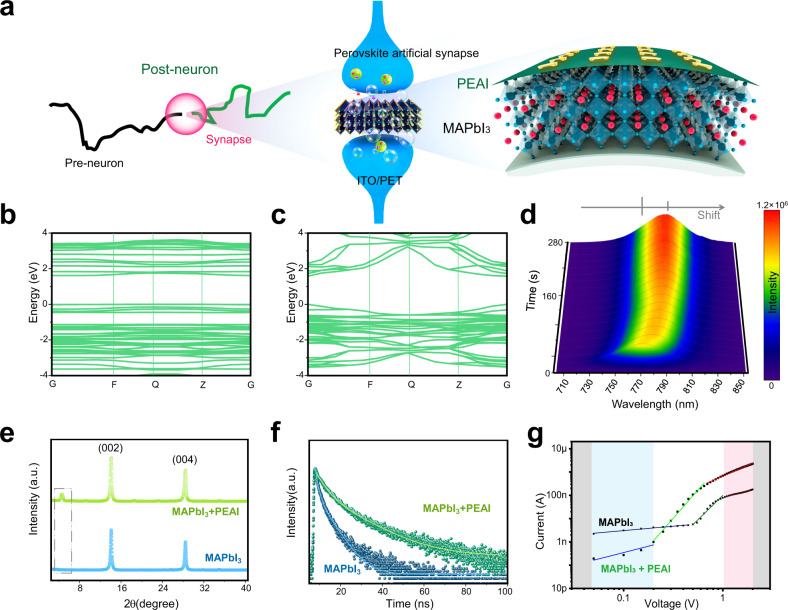
Concepts and materials. **a** Schematic diagrams of biological neuron/synapse and perovskite artificial synaptic device. **b**, **c** Energy band calculation of MAPbI_3_•n(MA) and MAPbI_3_. **d** Result of in-situ PL test. **e** XRD patterns of MAPbI_3_ with/without PEAI passivation. **f** TRPL spectra of MAPbI_3_ with/without PEAI passivation. **g** SCLC characteristics of MAPbI_3_ with/without PEAI passivation.

At RT, the perovskite precursor dispersed in a low-boiling point solvent was spin-coated on a flexible substrate, then passivated using an organic halide salt, PEAI, to suppress the defects of the polycrystalline perovskite. The PEAI-passivated perovskite film was stacked on indium tin oxide/PET then used to construct artificial synaptic devices (Fig. 1a). The migration of halide ions in the perovskite film mimicked the process of information transmission in the synaptic cleft^20^.

### Preparation of the perovskite at RT (RT-PVK)

Using methylamine-ethanol solution (MA-EtOH) and acetonitrile (ACN) as a combined solvent, we developed a room temperature method produces high-quality polycrystalline perovskite films with ultra-high [00*l*]-preferred orientation. The detailed preparation scheme and process are shown in Supplementary Fig. 1. Driving by the reactivity of CH_3_NH_2_ as a sigma donor to Pb^2+^, nitrogen atoms with lone electron pairs in methylamine to interact with the [PbI_6_]^4−^ octahedron^30,31^ to form an alcohol-soluble metastable intermediate, MAPbI_3_•n(MA) (Supplementary Fig. 2a). The low boiling points of EtOH and ACN^32^ lead to a high evaporation trend, which drives MA molecules to escape from the lattice, then leads to a phase transition to tetragonal β-phase MAPbI_3_ (Supplementary Fig. 2b). Calculation of the Gibbs free energy showed that the transformation is spontaneous at room temperature (Supplementary Fig. 2c). A movie visualizes this transformation process (Supplementary Movie 1). This structural change affects the band gap between the two states (Fig.1b, c). We performed an in-situ photoluminescence (PL) study to demonstrate this change (Supplementary Fig. 3). The PL peak position gradually redshifts from the initial 768–786 nm during crystallization process, and the peak intensity increases continuously (Fig. 1d). The migration of the PL peak indicates that the band of the two states decreases from 1.62 to 1.58 eV, which is close to the band gap calculation result of DFT.

PEAI can assume two forms on the surface of 3D perovskite; i.e., PEAI crystal layer, alone^21^, or a crystallized two-dimensional (PEA)_2_PbI_4_ perovskite nanolayer at the film surface^33^. In X-ray diffraction (XRD) patterns (Fig. 1e), the diffraction peaks of the PEAI-treated perovskite are almost the same as those of the control film without PEAI; i.e., a [00*l*] interplanar structure, including (002) and (004) peaks at 14.1° and 28.4°. However, the PEAI-passivated film presents a characteristic peak of PEAI at 4.7°, indicating the dominant form of PEAI salt on the perovskite surface is the PEAI crystal itself, rather than the 2D perovskite PEA_2_PbI_4_.

A cross-sectional scanning electron micrograph (SEM) image (Supplementary Fig. 4) demonstrates that the perovskite layer was ~720 nm thick, covered with a very thin PEAI passivation layer. Top-view SEM images of the control perovskite film without PEAI treatment present a homogeneous and pinhole-free surface (Supplementary Fig. 5a, b). However, perovskite film is uneven, with numerous grain boundaries. AFM measurements shows a root mean squared roughness *r*_RMS_ of 14.2 nm (Supplementary Fig. 5c). After PEAI passivation, the exposed surface of perovskite is completely covered (Supplementary Fig. 5d, e); the film is more uniform and smoother than the untreated surface, the grain boundaries are no longer obvious, and *r*_RMS_ decreases to 2.16 nm; these changes indicate that the passivation effect of PEAI improves the morphology of the perovskite film.

X-ray photoelectron spectroscopy (XPS) confirms the chemical structure on the surface of the perovskite layer. The binding energy *E*_b_ of Pb *4f*_5/2_ and Pb *4f*_7/2_ are reduced in the perovskite film that was treated with PEAI. Peaks at 136.1 and 141 eV disappears after treatment with PEAI; this result (Supplementary Fig. 5g) indicates that undercoordinated Pb^2+^ interacts with I^–^ in PEAI to passivate the defects efficiently^21,34^ Also, the I *3d* peak increases by the passivation (Supplementary Fig. 5h), indicating the presence of abundant iodide on the perovskite surface and that the iodine vacancy can be filled.

The absorption edge of the perovskite layer before and after passivation differed little; this result suggests that the presence of PEAI can improve the absorption intensity but does not change the absorption cutoff edge of perovskite film (Supplementary Fig. 6). The optical bandgap can be calculated using Tauc plots to be 1.58 eV^35^. Passivation of the perovskite layer increased its photoluminescence intensity; this change indicates a decrease in the number of trap states, and increase in the quality of the PEAI passivated perovskite films (Supplementary Fig. 6). Time-resolved photoluminescence (TRPL) measurements demonstrated that the decay time of the perovskite layer was obviously prolonged by passivation (Fig. 1f). These results suggest that PEAI-passivation of the perovskite films increases their charge-carrier lifetime and decreased their number of defects.

To estimate the trap density, space-charge-limited current (SCLC) measurements were performed on the perovskite devices before and after passivation. Three regimes were observed: namely Ohmic, trap-filled limit, and Child’s (Fig. 1g), where the trap-filled limit voltage *V*_*TFL*_ declined from 0.46 to 0.21 V after passivation. Therefore, calculated trap density using the following equation^36^: *n*_trap_ = 2εε_0_*V*_TFL_/(*eL*^2^) was 3.14 × 10^15^ cm^−3^ in pristine MAPbI_3_ and 1.43 × 10^15^ cm^−3^ in passivated MAPbI_3_.

### Neuroplastic regulation of RT-PVK artificial synapses

To mimic biological synapses, a perovskite artificial synapse with multilayer architecture of PET-ITO/MAPbI_3_/PEAI/Au was developed (Supplementary Figs. 7, 8). When a string of excitation signals is applied to the top electrode (TE, Au) of the device by the test system (Supplementary Fig. 9), the current signal collected at the bottom electrode (BE, ITO) of the device reaches its maximum at the peak amplitude.

To verify the repeatability of the device, we fabricated 10 chips (C1, C2, C3, …, C10) using the same material and process. Each chip held many dots of artificial synaptic devices (Supplementary Fig. 10). One device was randomly selected from each fresh chip and the *I*–*V* characteristics of the 10 devices were obtained. All 10 devices show similar hysteresis behaviour with switching ratios > 10^3^ (Supplementary Fig. 11). We ran 50 cycles of *I*–*V* tests on our devices in darkness. Except for the first three cycles, the device exhibits good cycle stability (Supplementary Fig. 12a). The same hysteresis behaviours were observed 5 h later (Supplementary Fig. 12b). The stable and repeatable *I*–*V* characteristic curve indicates that the hysteresis during the initial scan is not a result of preprocessing^37^, but is a result of migration of charge-carrier ions in the device^38^.

The hysteresis can be explained by the time-dependent ions migration under the electric field^39^. During positive-bias scanning, cations or vacancies move to ITO and accumulate on the side, so it becomes N-doped, while anions migrate to the Au side and form P-doped perovskite layer (Supplementary Fig. 13a). The perovskite film forms a P-I-N homojunction structure in polarization and a built-in electric field opposite to the applied voltage. Negative-bias scanning can flip the P-I-N structure to N-I-P by forcing ions or vacancies to drift in the opposite directions than in positive-bias scanning (Supplementary Fig. 13b).

We further explored the stability and uniformity of perovskite synaptic device (Supplementary Fig. 14). After exposure to air for 200 h, the PEAI-passivated device retains >73% of its initial conductance, and dose not decay much more afterward. After 240 h, five PEAI-passivated devices were randomly selected from the chip that had been exposed to the environment. Although the on-off ratio decreases after 240 h, all five samples retains similar hysteresis behaviour to those of fresh device (Supplementary Fig. 14b). The conductance and *I*–*V* curves prove the stability and uniformity of our passivated devices in ambient conditions.

**Fig. 2:**
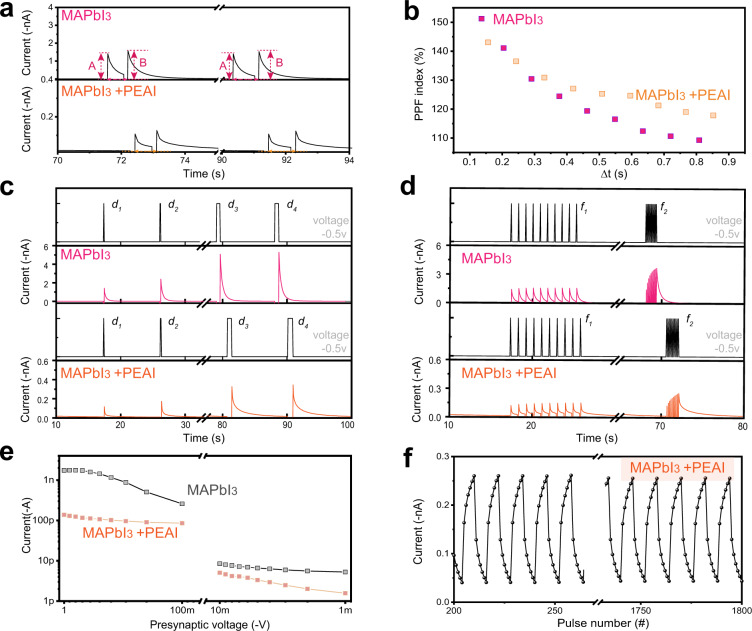
Short-term plasticity in RT-PVK artificial synapses. **a** EPSC triggered by a pair of spikes (−0.5 V, 68 ms) in perovskite artificial synapse with/without PEAI passivation. **b** PPF index versus time interval in perovskite artificial synapse with/without PEAI passivation. **c** SDDP behaviour of the device. EPSC triggered by consecutive spikes with different durations (*d*_1_ = 68 ms, *d*_2_ = 136 ms, *d*_3_ = 545 ms, *d*_4_ = 615 ms) and the same voltage amplitude (−0.5 V) in RT-PVK artificial synapse with or without PEAI passivation. Black: voltage spikes; pink and orange: response currents of the devices. **d** SRDP behaviour of the device. Frequencies *f*_1_ = 1.06 Hz, *f*_2_ = 6.39 Hz. Each group consisted of 10 spikes (−0.5 V, 68 ms) **e** EPSC triggered by spikes at varied voltages (−1 to −1000 mV). **f** Cycles of potentiation and depression in perovskite artificial synapse with PEAI passivation.

The device showed many responses that mimic the behaviors of biological neurons. Halide ions in the perovskite migrate under the action of an external electric field, and thereby increase the conductance of the active layer; this phenomenon is similar to the transmission of information between neurons. When RT-PVK synapse receives two consecutive presynaptic spikes in a short interval Δ*t*, the second postsynaptic response *I*_B_ is increased, and larger than the first one *I*_A_ (Fig. 2a and Supplementary Fig. 15); this phenomenon is called paired-pulse facilitation (PPF), which is a typical form of short-term synaptic plasticity^40^. The PPF index^41^ demonstrates that the passivated perovskite layer has a slow attenuation (Fig. 2b). As a result, the perovskite synaptic device with PEAI passivation has a small current peak and a relatively slow attenuation.

The change of synaptic weight is determined by the halide ions such as I^−^ migrate in perovskite. Therefore, the synaptic plasticity can be regulated by changing the form of input pulse stimuli (e.g., number, duration, amplitude) to propel halide ions. As the duration of the pulse was gradually increased from 68 to 615 ms, postsynaptic current increased accordingly due to increase in the number of halide ions that move (Fig. 2c). Spike-rate-dependent plasticity (SRDP) is thought to be an information transmission rule in the brain^39,42^. The artificial synaptic devices can achieve this function (Fig. 2d). When the interval between voltage spikes is 0.942 s (i.e., the spike frequency *f*_1_ = 1.06 Hz), the interval between the two spikes is long enough, so the drifted ions can diffuse back to their initial distribution, and the device can return to its initial state before the next spike arrives. As a result, the EPSC dose not change significantly after voltage spike stimulation at different times. When the interval between voltage spikes is decreased to 0.156 s (i.e., *f*_2_ = 6.39 Hz). In this case, the EPSC increases obviously. This result occurs because the next spike is applied before the back-diffusion of ions had been completed^39^, so the newly-applied spike causes a net increase in the quantity of diffused ions, and thereby increases the polarization current through the device.

Decrease in the pulse amplitude, the current through the passivated artificial synapse showed better linearity in response to voltage (Fig. 2e), and its residual ratio decreased significantly compared with the control group at voltage magnitudes of −0.1 ~ −1 V (Supplementary Fig. 16). This change indicates increased uniformity of voltage modulation capability. Furthermore, to quantify the endurance of the device, six positive and negative pulses (−0.5 V, 0.1 V, 68 ms) were applied continuously. The current shows only slight change after 1800 pulses. This result indicates that PEAI treatment a yielded stable and durable device (Fig. 2f).

### Dynamics of ion migration and energy consumption

**Fig. 3:**
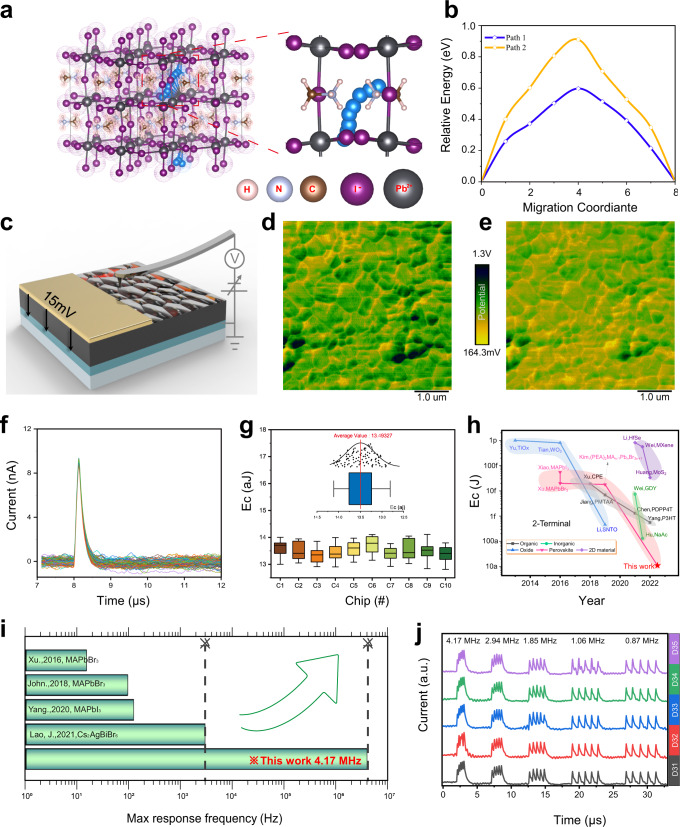
Dynamics of ion migration and Ultra-low energy consumption. **a** Path 1: vertical movement (blue balls) along PbI_6_ octahedral layer, and Path 2: lateral movement (blue balls) through the gap of MA^+^. **b** Corresponding activation energies for Path 1 and Path 2. **c** Schematic illustration of i-KPFM. **d** Surface potential profile before application of 15 mV bias. **e** Surface potential profile during application of 15 mV bias. **f** EPSC triggered by same spikes (15 m V, 100 ns) for 100 devices (10 devices on each of 10 chips). **g** Statistics of energy consumption of 100 devices. **h** Comparison of energy consumption *E*_c_ per synaptic event for different types of 2T artificial synapses. **i** Comparison of the maximum response frequency of perovskite artificial synapses. **j** SFDP behaviour of five identical devices (D31–D35) at different frequencies (4.17, 2.94, 1.85, 1.06, 0.87 MHz) under five consecutive spikes (15 mV, 100 ns).

Iodide in perovskite has a low migration activation energy, which is beneficial to the development of ultra-low energy consumption artificial synapses^43^. Two possible migration paths were obtained by DFT calculation, which are vertical movements along the PbI_6_ octahedral layer (Path 1, Fig. 3a) and lateral movements through the gap of MA^+^ (Path 2, Supplementary Fig. 17). Relative energy distributions (Fig. 3b) result shows the lowest activation energy of 0.597 eV for vacancy-assisted diffusion of iodide ions through Path 1. This suggests that extremely low energy is sufficient to drive I^−^ migration. Employed in operando Kelvin probe force microscopy (i-KPFM) to examine the surface potential profile under a bias voltage (Fig. 3c), we found that I^−^ can migrate even at a low voltage of 15 mV. The film’s surface potential is 0.721 V when no voltage is applied (Fig. 3d). However, after applying a bias voltage of 15 mv, the surface potential drops to 0.613v (Fig. 3e). The reason is that I^−^ with negative charges migrates to the top under the action of voltage and causes the decrease in the surface potential. A more visual proof by time-of-flight secondary ion mass spectrometry (TOF-SIMS) show that there is a significant upward migration of I^−^ after 500 spikes of 15 mV (Supplementary Fig. 18). Under a 15 mV spike with width = 100 ns, we tested a series of devices with different sizes (Supplementary Fig. 19). The statistics of device energy consumption (Supplementary Fig. 20) indicate that the current and energy consumption are positively correlated with device size. The energy consumption can be reduced to ~13.42 aJ /synaptic event in a device with diameter 100 μm. To avoid contingency, we repeatedly tested the EPSC behaviour of synaptic devices by using these spikes (15 mV, 100 ns) on 100 devices (Fig. 3f). Then we calculated the energy consumption of each device, and obtained a boxplot to measure the level of dispersion within an energy consumption dataset. The distribution of energy consumption is narrow with small difference in the energy consumption between the chips of 12.81–14.11 aJ per synaptic event (Fig. 3g). The average energy consumption of 100 devices was 13.5 aJ. It is the lowest energy consumption ever recorded for a perovskite-based artificial synaptic device (Fig. 3h and Supplementary Table 1).

The ultralow energy consumption is a result of rapidly-crystallized RT perovskites with ordered lattice packing that reduces the number of disordered electronic states, and improves charge transport property^23^. Furthermore, PEAI can passivate defects and suppress ion migration. AFM measurements were conducted to quantify perovskite film’s surface height. The grain boundaries are the deepest place on the surface of the RT-PVK (Supplementary Fig. 22a). PEAI includes numerous phenethylamine groups. Characterization of the distribution of their characteristic (C_6_H_5_^−^) groups was characterized using TOF-SIMS indicates that PEAI is dispersed both on the surface and in the bulk of perovskite. This is due to PEAI salt is more easily deposited at the grain boundary and diffuses into the perovskite bulk^21,44,45^ (Supplementary Fig. 22c–e). The undercoordinated Pb^2+^ can coordinate with the I^–^ anion of PEAI to fill the iodine vacancy. The –NH_3_^+^ group of PEAI can bind with I^–^ of PVK to occupy the cation vacancy, and thereby passivate defects and suppress ion migration in the bulk film^34^. It reduces the number of defects available for ions migration (Supplementary Fig. 23), resulting in a small EPSC response and increasing the energy efficiency.

At this low energy consumption, devices retain synaptic properties and exhibit dynamic responses at high working frequency (4.17 MHz). This is the fastest response frequency ever reported for a perovskite artificial synapse (Fig. 3i). Repeated experiments confirmed the stability of the device under this frequency (Fig. 3j). In addition, PPF behaviour was obtained at ultrahigh-frequency mode (Supplementary Fig. 24). In contrast to low-frequency mode, the EPSC is generated by diffusion currents that are lagged relative to the stimulation voltage and dominated by the built-in electric field (Supplementary Movie 2). In ultrahigh-frequency mode, the plasticity superposition behaviour of synapses can be achieved by synchronization with the stimulus (Supplementary Fig. 21).

The mechanical flexibility was assessed by bending with a radius curvature of 4.5 mm (Supplementary Fig. 25). *I*–*V* curves of passivated device varied only slightly under continuous bending cycles, i.e., the mechanical characteristics are reliable. The conductance in the passivated device remained stable at ~7 nS, but declined from 80.4 to 66.3 nS in the unpassivated sample. Under stimulation by two short spikes (15 mV, 100 ns), the PPF index for passivated device remained relatively stable after 2000 bending cycles, but decayed continuously in the unpassivated device. Compared with other artificial synapses that use perovskites (Supplementary Table 2), this passivated device shows advances in information processing speed, even during bending. These results suggest a reliable mechanical flexibility and synaptic plasticity for perovskite synapse with PEAI passivation even during in high-speed computing.

### Logic, spatiotemporal correlation and learning

**Fig. 4:**
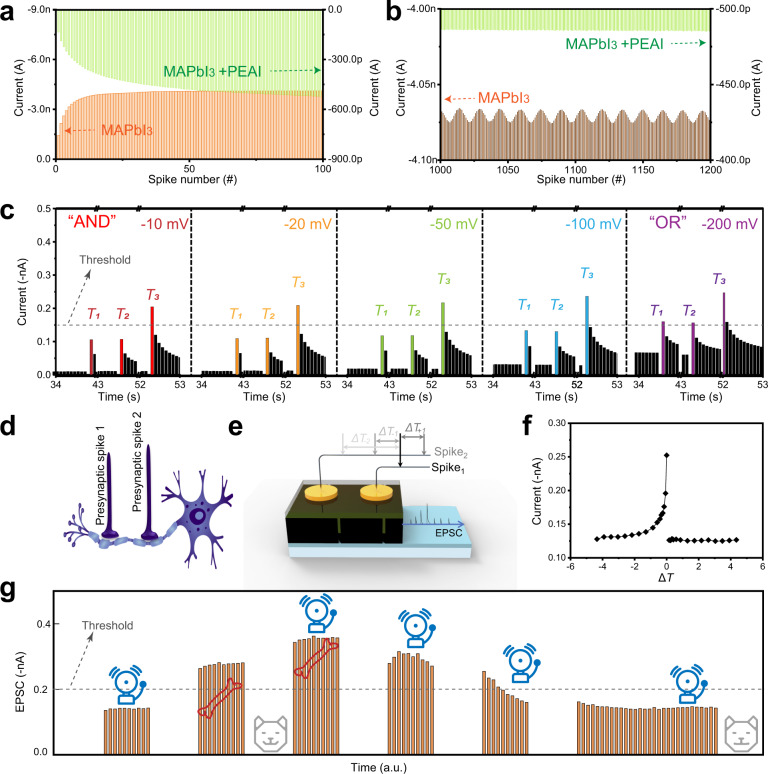
Logic, spatiotemporal correlation, and learning in RT-PVK artificial synapses. **a** EPSC triggered by 100 consecutive spikes (−0.5 V, 68 ms) in perovskite artificial synapse with/without PEAI passivation. **b** EPSC triggered by 2000 consecutive spikes (−0.5 V, 68 ms) in perovskite artificial synapse with/without PEAI passivation. **c** Logical operations in perovskite artificial synapse with PEAI passivation. **d**, **e** Schematic diagram of spatiotemporal-correlation signal-processing in organisms and RT-PVK artificial synapses. **f** EPSC versus interval Δ*T* between spike 1 and spike 2 (Δ*T* < 0: spike 2 after spike 1; Δ*T* > 0: spike 1 after spike 2). **g** Conditional reflex learning in perovskite artificial synapse with PEAI passivation.

When pulse excitations were applied continuously, the current gains differed between the passivated and unpassivated devices. After 50 pulses, the current amplitude of the passivated device increased obviously, whereas the current through the unpassivated device showed almost no change (Fig. 4a). The passivated device still showed good gain after 100 consecutive excitations. The current through the two devices also behaved differently after 1000 consecutive pulse excitations (Fig. 4b). After initial stimulation using 1000 pulses, an additional 200 consecutive pulses were applied to the device to observe its stability under a mass of continuous pulse. After 1200 pulses, the MAPbI_3_ device without passivation demonstrate oscillating current, because persistent voltage stimulation results in substantial ion migration, oxidation of halides at the anode, and reduction of potential components (e.g., methyl ammonium) at the cathode^46^; the result is electrolysis of the perovskite and formation of new non-perovskite minerals with different electrical properties from the original MAPbI_3_. In contrast, the passivated device displays a relatively constant current response to the additional 200 pulses. This result occurs because passivation decreases the number of removable defects and weakens the electrolytic degradation of perovskite.

Then the input programming of multiple TEs at a low-amplitude voltage were implemented (Supplementary Fig. 26). When the reading voltage (*V*_re_) is continuously increased from −20 to −200 mV, the baseline current also increased, and the peak current eventually surpasses −0.15 nA. Essential logic operations can be realized by adjusting the amplitude of the *V*_re_ to control the peak value of the post-synaptic current. At a low voltage, the threshold (−0.15 nA) can only be reached by near-simultaneous input from multiple TEs, similar to a logical “AND”. As the *V*_re_ increases to −200mV, the threshold can be broken even under the input of a single electrode, similar to a logical “OR”. In a biological system, a single strand of dendrite to one postneuron (Fig. 4d), which can connect two presynaptic with spatiotemporally information; Fig. 4e schematically describes the implementation of this function on the RT-PVK artificial synapse. When presynaptic spike_1_ was applied at time *t*_spike1_, and presynaptic spike_2_ was applied at *t*_spike2_ on another TE, with an interval Δ*T* = *t*_spike2_ *−* *t*_spike1_ between them, the ion migration caused by spike_2_ can be spatiotemporal superimposed with spike_1_. It was greatest, almost twice the initial current, when Δ*T* = 0 ms (Fig. 4f).

The inputs of the two TEs are regarded as “food” and “bell” to realize the rules of associative (Pavlovian) learning. Here, the pulse excitation with the larger amplitude is used as the “food” stimulus, whereas the “bell” stimulus is trained (Fig. 4g and Supplementary Fig. 27). When the postsynaptic current breaks through the set threshold (−0.2 nA), it means that saliva is produced under the stimulation of “food” or “bell”. After several epochs of auxiliary training, the “bell” stimulus can increase the magnitude of the postsynaptic currents. These results suggest that associative learning can be implemented in the passivated perovskite artificial synaptic device.

### Bionic muscle fatigue model

**Fig. 5:**
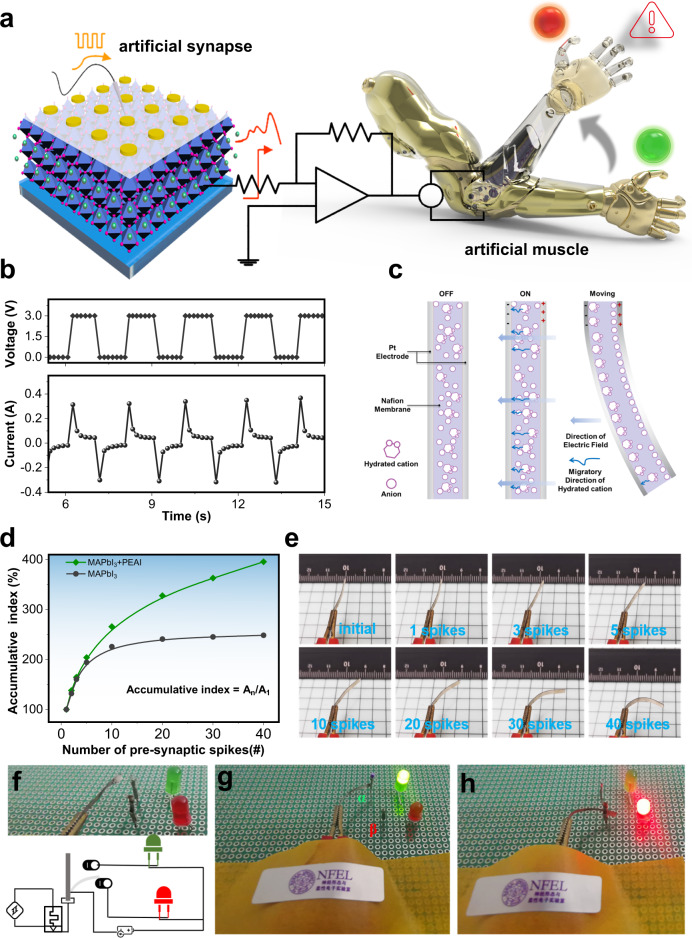
Neuromuscular electronic system. **a** Configuration of perovskite artificial synapse and neuromuscular electronic system. **b** Current response in IPMC at an operating voltage of 3 V. **c** Structure and motion mechanism of IPMC artificial muscle. **d** Accumulative index as triggered by consecutive presynaptic spikes on perovskite artificial synapse. **e** Digital images of the IPMC artificial muscular according to EPSC as triggered by different numbers of consecutive spikes. **f** Digital photos and diagrams of neuromuscular logic circuit of muscular-fatigue warning. **g**, **h** Results of muscular-fatigue early warning model.

Multiple accumulative processing ability of neuromorphic devices is very important in the neuromorphic information-processing paradigm^47^. Formation of an environmental stimuli-responsive artificial efferent nerve or neuromuscular system^48^ remains a challenge. As a proof of concept, we fabricated a neuromuscular system by combining a room-temperature perovskite artificial synapse and electrochemical artificial muscles (Fig. 5a). Here, the artificial muscle is an ionic polymer-metal composite (IPMC), consisting of two Pt electrodes on both sides and a Nafion membrane in the middle. Compared with other soft actuators^49^, IPMC can be operated at relatively low voltage^50^, approximately 2–3 V. The resultant current in IPMC is around 0.33 A at 3 V (Fig. 5b). Driven by electric field, hydrated cations in the Nafion membrane migrate to the cathode (Fig. 5c), resulting in asymmetric swelling of the Nafion membrane, and the deflection to the anode^51–53^ (Supplementary Fig. 28). Different numbers of presynaptic spikes were applied to the top electrodes of the perovskite synapse as sensory information, and the bottom electrodes were connected to electrochemical artificial muscles by accessory circuits (Supplementary Fig. 29) to construct a neuromuscular system. A passivated perovskite synapse showed better accumulative and harmonic effects than an unpassivated counterpart. After 30 consecutive spikes, accumulative index reached 362.9% in a passivated perovskite synapse (Fig. 5d), but only 245% in the unpassivated perovskite layer.

The EPSC peak of a passivated synapse varied with the number of presynaptic spikes. Under fewer than three stimuli, the synaptic weight change of the perovskite synapse was relatively low with accumulative index <150%, and the artificial muscle remained in an inactive state, unable to achieve neuromuscular system excitation (Fig. 5e). When increased numbers of repetitive events frequently stimulated the synapse, accumulative index exceeded the threshold (150%), and the artificial muscle was directed to the corresponding amplitude of movement. Electrochemical deflection of artificial muscles at different levels of excitation was shown (Fig. 5e). Increase in EPSC caused increase in the deflection of the electrochemical artificial muscle. The maximum deflection angle exceeded 90° after 40 consecutive spikes.

In biological tissues, an excess of continuously excitatory stimuli is detrimental, and results in muscle fatigue^54^. Flexible artificial muscle actuator is an important component of intelligent and interactive soft robot system^55^. At present, these actuators cannot sense fatigue. Cyclic load under long-term bending and large curvature bending can easily induce device fatigue and microscopic damage, which lead to decreased efficiency and dynamic changes of the actuator, and even to breakdown^56^. These responses may cause hidden dangers to the safe operation. By introducing a system to warn of neuromuscular fatigue, the fatigue risk of the actuator is indicated, and this response can help to improve the reliability and safe operation. To avoid this effect, we designed a neuromuscular fatigue warning system (Fig. 5f). Under normal excitation, the electrochemical artificial muscle deflected at a small angle and connected terminal α, so the system activated a green light (Fig. 5g); in contrast, under excessive excitation, the artificial muscle deflected enough (>90°) to connect terminal β, so the system activated a red light to indicate alarm, to realize early warning of muscle fatigue (Fig. 5h). These results is potentially useful as a functional module in future neurorobots and biological system^29^.

## Discussion

A flexible perovskite synaptic device was fabricated at RT by using a volatile solvent without any thermal annealing or post-treatment. The perovskite artificial synapse emulates essential synaptic functions, including logical operations, temporal and spatial rules, and associative learning. Passivation of the perovskite reduced its density of defects and increased its optoelectronic properties, and yielded an artificial synaptic device that had attojoule-level energy-efficient properties with an ultra-fast response frequency of up to 4.17 MHz. After 2000 bending cycles, the device’s electrical characteristics remained reliable. As a proof of concept, we fabricated an artificial neuromuscular system by combining a room-temperature perovskite artificial synapse with electrochemical artificial muscles, and successfully achieved the muscular-fatigue warning system. The findings here may provide a simple protocol to manufacture synaptic devices, and may provide new insights to guide the development of artificial synapses for use in neuromorphic bioelectronics and neurorobots.

## Methods

The Supplementary Information displays more experimental details.

### Materials preparation

Methylamine (30–33% in ethyl alcohol), isopropyl alcohol (99.5%), and acetonitrile (99.5%) were purchased from Aladdin. Methylammonium iodide (99.5%), lead iodide (99.99%), and 2-phenylethanamine iodide (99.5%) were purchased from Xi’an Polymer Light Technology Co., Ltd. All these reagents were used without further purification.

### PVK ink fabrication

MAPbI_3_ solution (1.1 mol/L) was prepared by dissolving MAI and PbI_2_ into a mixture of methylamine and acetonitrile (volume ratio = 3:2) at molar ratio of 1:1, then stirred for 1 h at RT (Supplementary Information Note 1). PEAI solution (5 mg/mL) was prepared by dissolving PEAI in IPA solution then letting it stand for 2 h to dissolve completely.

### Device fabrication

ITO substrate was cleaned by sequential ultrasonic treatment in deionized water, acetone and IPA for 15 min each. The substrate was dried using blown N_2_ gas, then treated by UV-zone for 20 min to increase wettability. The device was fabricated using a dynamic solution-processing method: 100 µL of as-prepared MAPbI_3_ solution was dropped on a steadily-spinning substrate and spin-coated at 4000 rpm for 60 s. Then 70 µL PEAI solution was dropped on the MAPbI_3_ film and spin-coated at 4000 rpm for 30 s. All procedures were conducted in an N_2_-filled glove box at RT. Au electrode was developed by thermal evacuation through a specify shadow mask (Supplementary Information Note 2).

### Reporting summary

Further information on research design is available in the Nature Portfolio Reporting Summary linked to this article.

## Supplementary information

Supplementary Information

Description of Additional Supplementary Files

Supplementary Movie 1

Supplementary Movie 2

Reporting Summary

## Data Availability

The source data generated in this study are provided in the ‘Source Data’ file. Source data are provided with this paper.

## Source data

Source Data
