# Supplementary material for: A bioinspired flexible neuromuscular system based thermal-annealing-free perovskite with passivation
Source: Nat Commun. 2022 Dec 2;13:7427. doi: 10.1038/s41467-022-35092-w (PMC9718817; doi:10.1038/s41467-022-35092-w)
Supplement: Supplementary file 1 — Supplementary Information [file 41467_2022_35092_MOESM1_ESM.pdf]

Supplementary Information

A Bioinspired Flexible Neuromuscular System Based Thermal-Annealing-Free Perovskite with Passivation

Jiaqi Liu[1,2,3#], Jiangdong Gong[1,2,3#], Huanhuan Wei[1], Yameng Li[1,4], Haixia Wu[4], Chengpeng Jiang[1,2,3], Yuelong Li[1*], Wentao Xu[1,2,3*]

[1]Institute of Photoelectronic Thin Film Devices and Technology of Nankai University; Solar Energy Research Center of Nankai University; Key Laboratory of Photoelectronic Thin Film Devices and Technology of Tianjin; Engineering Research Center of Thin Film Photoelectronic Technology, Ministry of Education, #38 Tongyan Road, Jinnan District, Tianjin 300350, P. R. China.

[2]Shenzhen Research Institute of Nankai University, Shenzhen 518000, China

[3]Smart Sensing Interdisciplinary Science Center, Nankai University, Tianjin 300350, China

[4]College of Chemical and Pharmaceutical Engineering, Hebei University of Science and Technology, Shijiazhuang 050018, Hebei, China.

#These authors contribute equally (J.L. and J.G.).

*Correspondence and requests for materials should be addressed to W.X. (wentao@nankai.edu.cn) or Y.L. (lyl@nankai.edu.cn).

Supplementary Methods

1.Artificial muscles fabrication.

Electrochemical artificial muscles were prepared by autocatalytic reduction plating. Firstly, a surface with certain roughness was formed by equal sandblasting treatment on both sides of a Nafion membrane. The Nafion film was sequentially treated in hydrochloric acid (2 mol/L), deionized water, hydrogen peroxide, and DI water, then immersed in a mixture of 250 mL $Pt(NH_3)_4Cl_2$ (1 wt%) and 20 mL $NH_3 \cdot H_2O$(18wt%) for 24 h. Then the Nafion membrane was transferred into 250 mL DI water, and then 40 mL of 5 wt% $NaBH_4$ as a reducing agent, was gradually dropped 20 times over a span of 4 h.

2.Characterizations.

AFM and i-KPFM images were obtained using A Bruker dimension icon microscope in tapping mode. XRD patterns were obtained using a Rigaku Ultima IV instrument. XPS was conducted using an ESCALAB 250Xi (Thermo Scientific). The curve of optical absorption was acquired from spectrophotometer (Cary 5000). Using the fluorescence spectrometer (Edinburgh FS5) to get PL and TRPL spectra. All electrical measurements were performed using a Keithley 4200A semiconductor parameter analyzer and a probe station in N_2 atmosphere in a glove box at room temperature.

3.DFT calculations.

We have employed the Vienna Ab Initio Package (VASP)[S39,S40] to perform all the density functional theory (DFT) calculations within the generalized gradient approximation (GGA) using the PBE[S41] formulation. We have chosen the projected augmented wave (PAW) potentials[S42, S43] to describe the ionic cores and take valence electrons into account using a plane wave basis set with a kinetic energy cutoff of 520 eV. Partial occupancies of the Kohn−Sham orbitals were allowed using the Gaussian

smearing method and a width of 0.05 eV. The electronic energy was considered self-consistent when the energy change was smaller than 10^{-5} eV. Geometry optimization was considered convergent when the force change was smaller than 0.03 eV/Å. Grimme's DFT-D3 methodology[S44] was used to describe the dispersion interactions. The Brillouin zone integral uses the surfaces structures of 2×2×1 monkhorst pack K-point sampling. The formation energies of different species (E_f) were computed using the chemical potentials for each constituent atom, determined by solving the set of linear equations describing the contributions from the starting species in the pristine. Finally, We define the formation energy per atom (E_f) of a compound, where the dopant

$$E_f = E_{total} - N_{Pb\mu Pb} - N_I\mu_I - N_C\mu_C - N_N\mu_N - N_H\mu_H \,, \tag{3}$$

where E_{total} is the total DFT energy of a given structure, and $\mu_{(Pb,I,C,N,H)}$ are the chemical potentials of the constituent atomic species. $N_{(Pb,I,C,N,H)}$ are the number of the constituent atomic species.

The free energy was calculated using the equation:

$$G = E_{ads} + Z_{PE} - T_S, \tag{4}$$

where G, E_{ads}, Z_{PE} and T_S are the free energy, total energy from DFT calculations, zero point energy and entropic contributions, respectively.

The Climbing Image-Nudged Elastic Band methods had been employed to calculate the I^- migration barriers in the structures.

Supplementary Notes.

1. Preparation of perovskite film at RT

The method to prepare perovskite films at room temperature (Supplementary Fig.1). 1.1mmol PbI_2 and MAI powders were mixed with 600µl EtOH solution containing 33% wt methylamine, then stirred at 400 rpm for 30 min at room temperature. This process dissolved most of the solids to yield a turbid solution. 400µl ACN was quickly added to the solution and the mixture was gently shaken; during this process, it quickly became clear. To ensure that all the solids dissolve evenly, the stirring was continued at a 400 rpm for 30 min to yield the perovskite precursor ink (PVK ink). The cleaned ITO was accelerated to 4000 rpm for spin coating, then 100 µL PVK ink was dropped on the centre of the ITO. ACN and EtOH have lower boiling points, so they can volatilize quickly and make the yellowish PVK ink turned to glossy black perovskite films in < 10 s. After the colour changed, the film was spun for another 50 s to ensure that the solvent had volatilized completely to leave perovskite film.

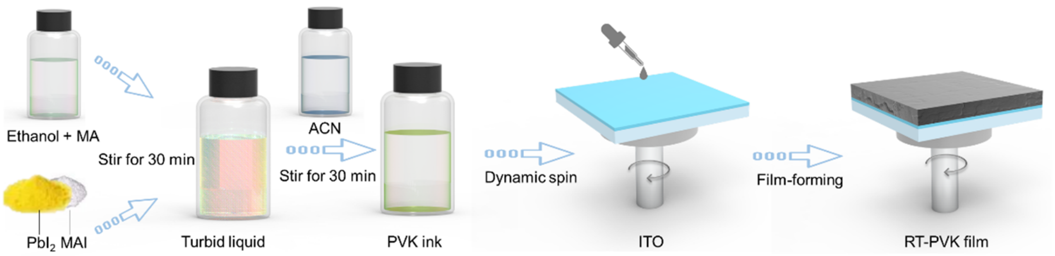

Supplementary Figure 1. **Preparation flow chart of perovskite film.** All steps were performed at room temperature (RT).

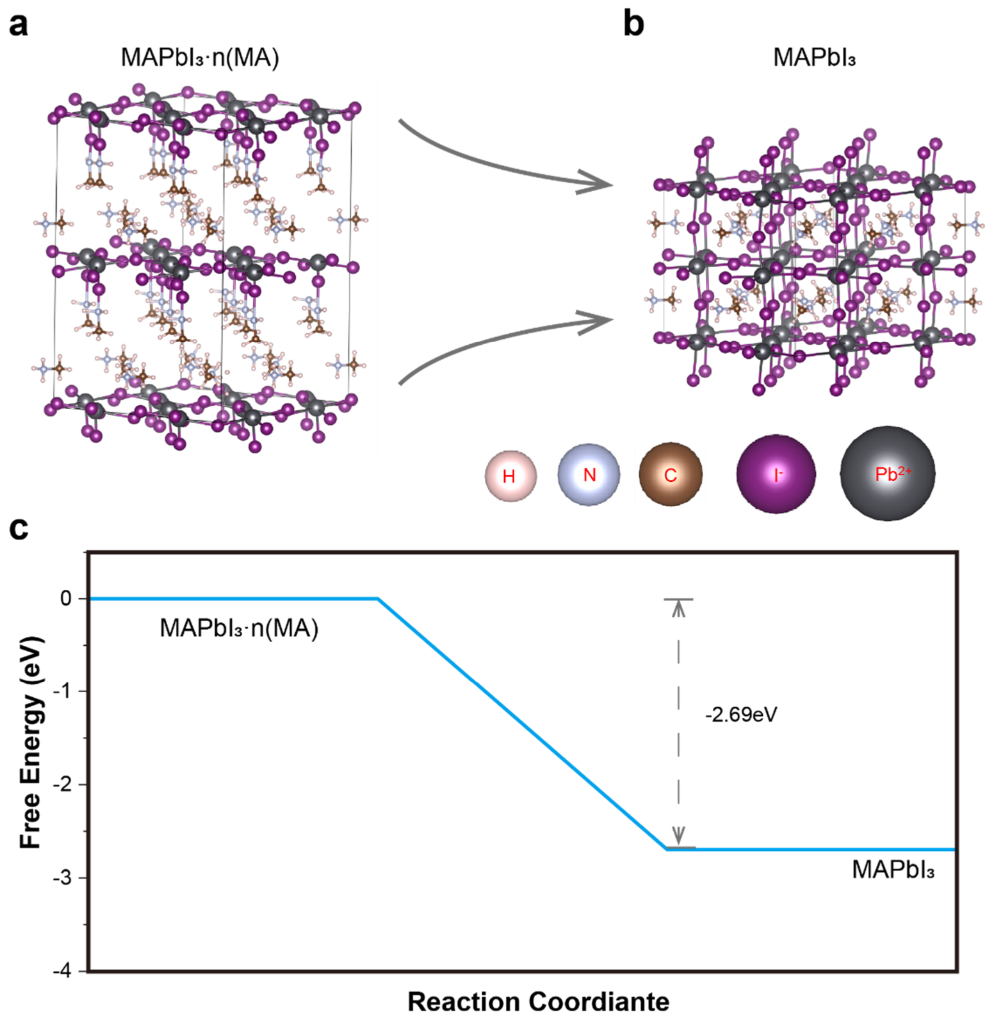

Supplementary Figure2. **Structural transformation during perovskite formation**. **a** Structure of metastable intermediate, $MAPbI_3$•n(MA). **b** Structure of final tetragonal β-phase MAPbI3.**c** Gibbs free energy calculations of $MAPbI_3$•n(MA) to $MAPbI_3$.

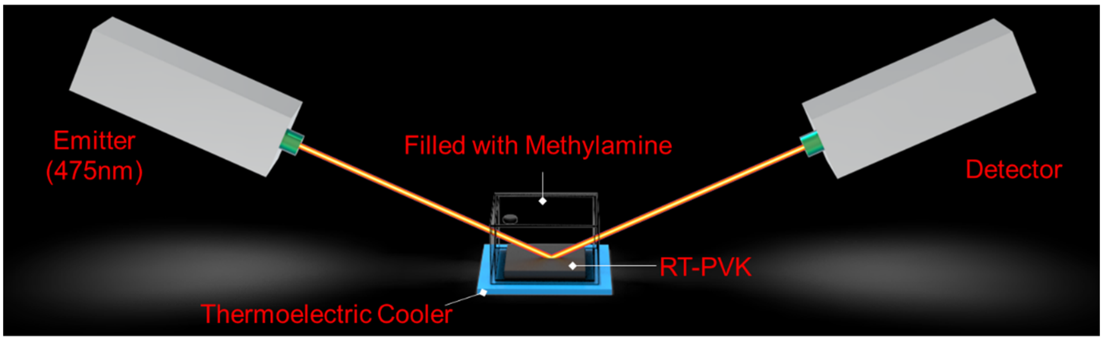

Supplementary Figure3. **Schematic of in-situ PL tracing during crystallization process.** To decelerate the crystallization process, the were placed samples in a transparent sealed environment filled with methylamine gas to increase saturation pressure and slow the evaporation PVK ink. A 25 °C constant-temperature cooling sheet was placed at the bottom.

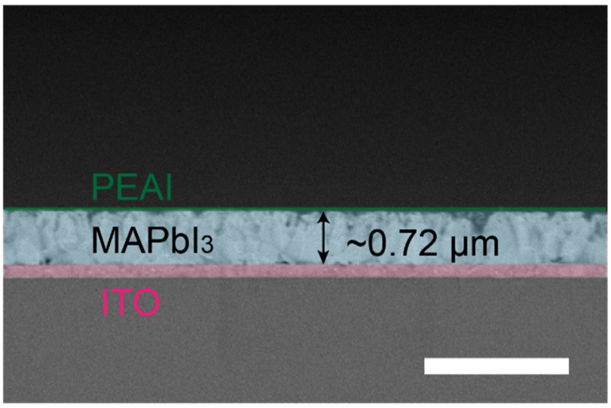

Supplementary Figure4. **Cross-sectional** Images of perovskite artificial synaptic device, scale bar : 2 μm

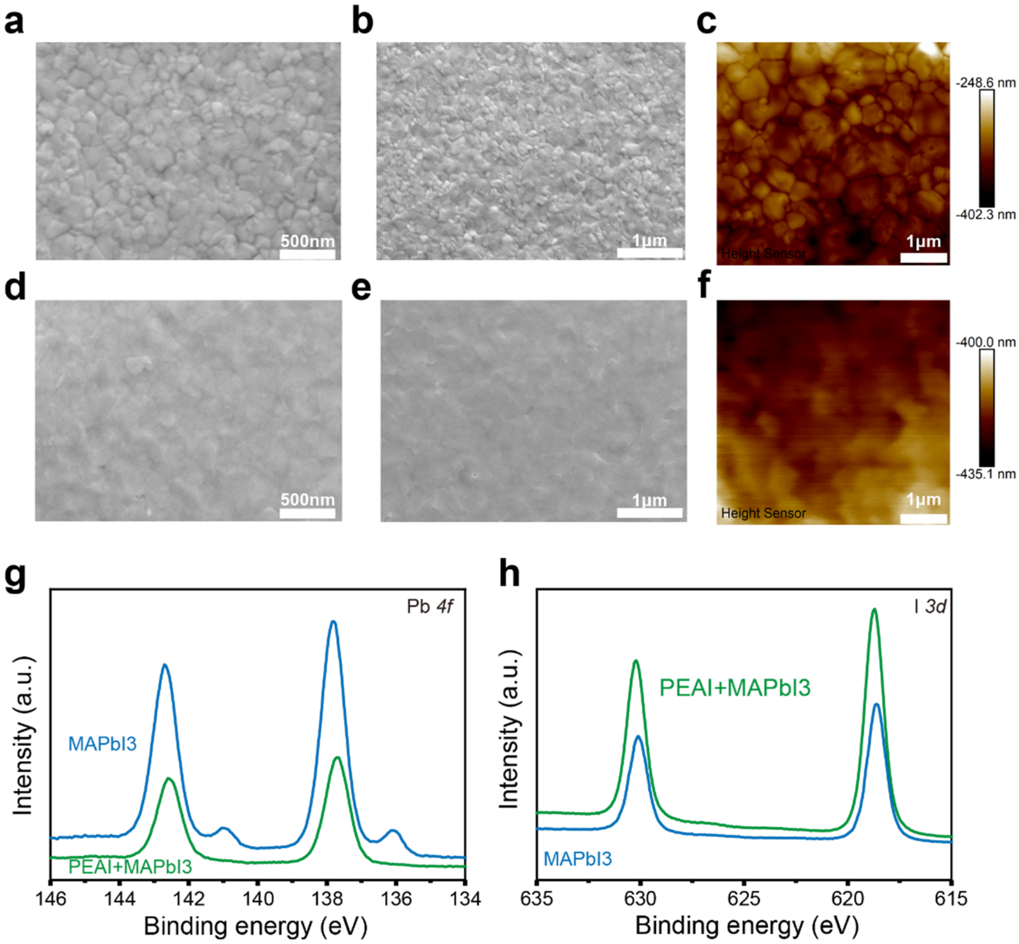

Supplementary Figure5. **Characterization of the films. a, b** Top view SEM of RT-PVK films without PEAI. **c,** AFM height images of RT-PVK films without PEAI. **d, e** Top view SEM of RT-PVK films with PEAI. **f,** AFM height images of RT-PVK films without PEAI. **g** Pb *4f* core energy level of MAPbI3 with and without PEAI passivation. **h** I *3d* core energy level of MAPbI3 with and without PEAI passivation.

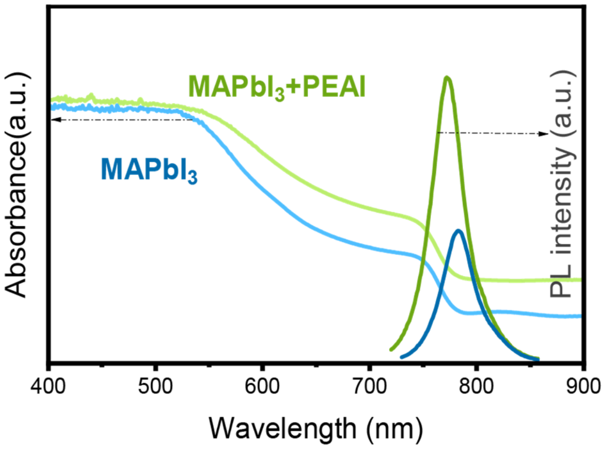

Supplementary Figure6. **Steady-state absorbance and photoluminescence of MAPbI3 with/without PEAI passivation.**

2. Fabrication of RT-PVK artificial synapses

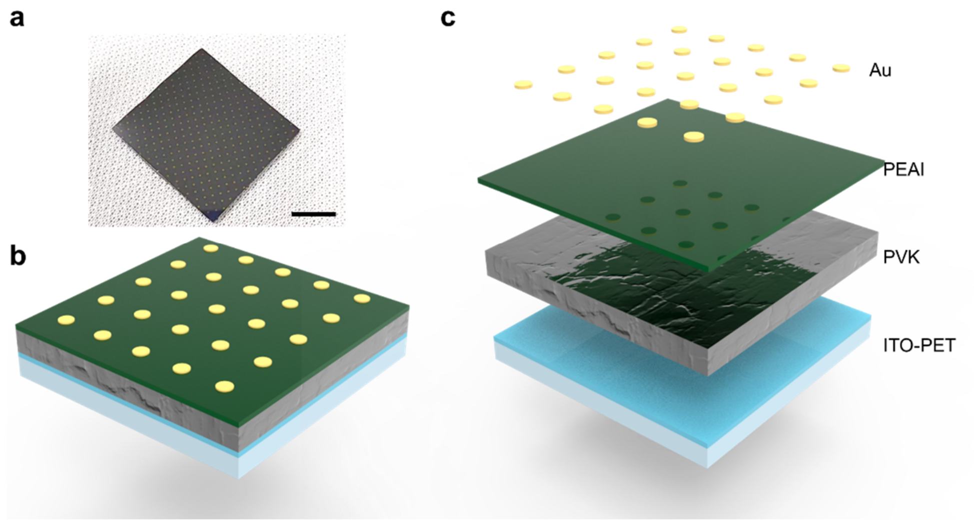

Supplementary Figure7. **Structure of RT-PVK artificial synapses . a** Optical photograph of RT-PVK artificial synapses, scale bar :1cm. **b** Schematic diagram of RT-PVK artificial synapses. **c** Exploded views of RT-PVK artificial synapses.

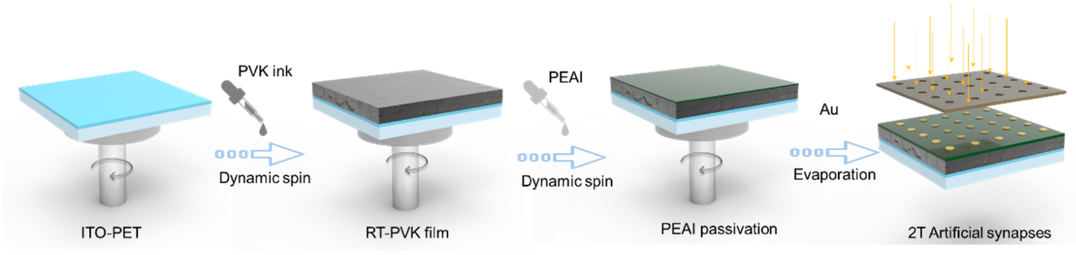

Supplementary Figure8. **Flowchart illustrating the construction of an artificial synapses.**

The device was prepared by spin coating and evaporation as the main processing methods. A schematic (Supplementary Fig.8) of device fabrication illustrates the process. 100 μl of as-prepared $MAPbI_3$ PVK ink was dropped on a steadily-spinning substrate and spin-coated at 4000 rpm for 60 s. Then 70 μl PEAI solution was dropped

on the $MAPbI_3$ film and spin-coated at 4000 rpm for 30 s. All procedures were conducted in an N_2-filled glove box at RT. Finally, a mask evaporation process was used to plate electrodes on the upper surface.

3. Test of RT-PVK artificial synaptic devices.

The signals were sent and read by a semiconductor analysis test system (4200-SCS) (Supplementary Fig.9a ,c). ITO is revealed when a small section of PEAI and perovskite is scraped away. The voltage signal emitted by 4200-SCS was applied using two probes, one connecting to the ITO and the other to the Au. The voltage after perovskite artificial synapses was transformed to a postsynaptic current similar to biological neural signals (Supplementary Fig.9b).

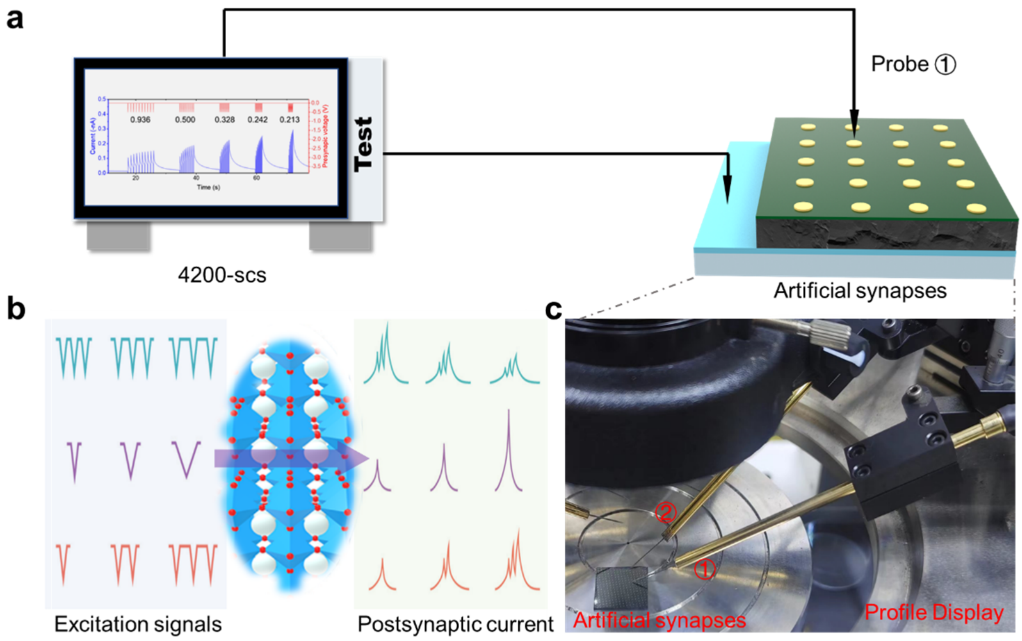

Supplementary Figure9. **Device test system. a** Schematic diagram of test device. **b** Schematic illustration of spikes dependent plasticity under trains of negative pulses (-0.5v). **c** optical photo of device test. Probes ① and ② were connected to Au and ITO of the artificial synapses, respectively.

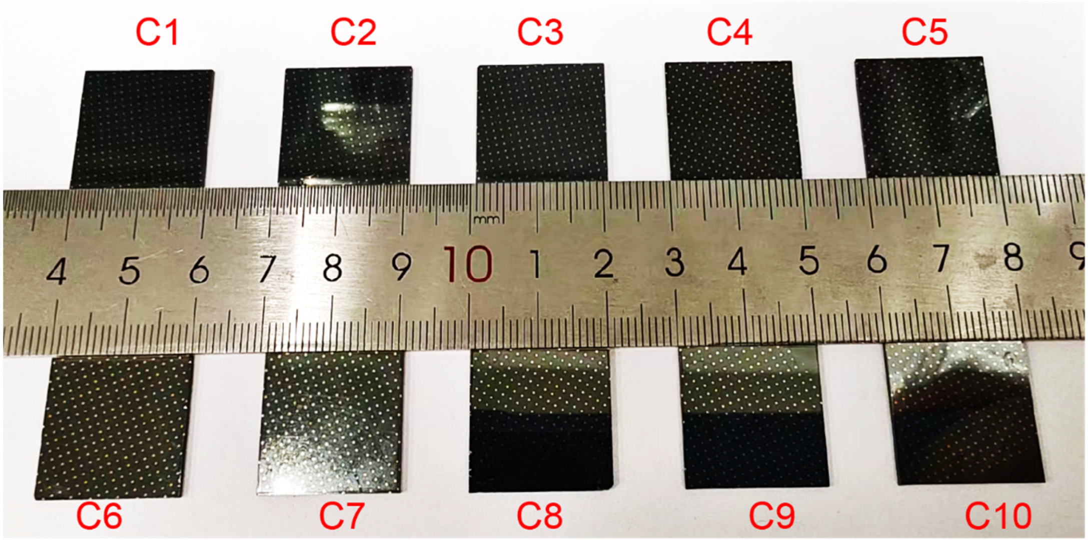

Supplementary Figure10. **Optical photograph of RT-PVK artificial synapse.** Optical photos of 10 chips (C1, C2, C3, …, C10) prepared by the same process.

4. *I-V* characteristics of RT-PVK artificial synapses

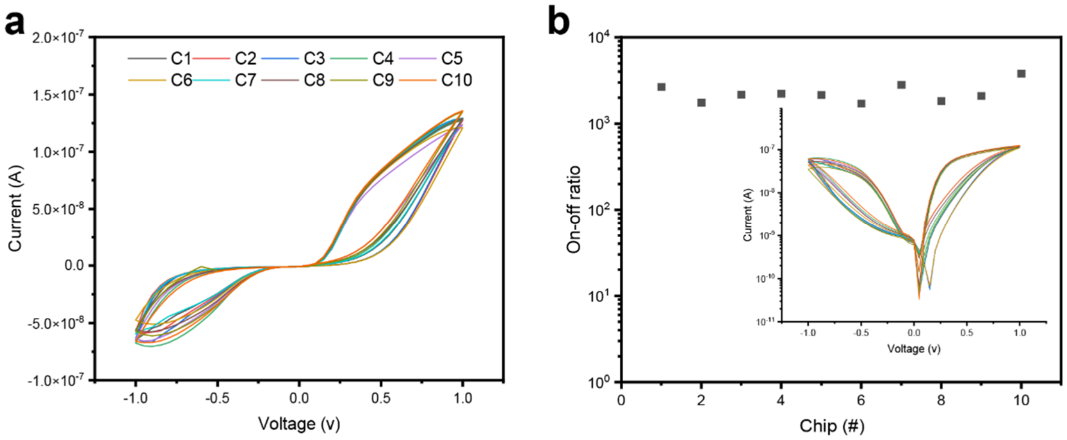

Supplementary Figure11. **Reproducibility test. a** *I–V* characteristics of fresh devices on different chips for the 1st cycle. Scanning direction was 0 V→ +1 V→ 0 V→ -1 V at 0.05 V·s^{-1}. **b** Statistics of the on-off ratio of different devices (inset: *I–V* characteristic on a semi-log scale).

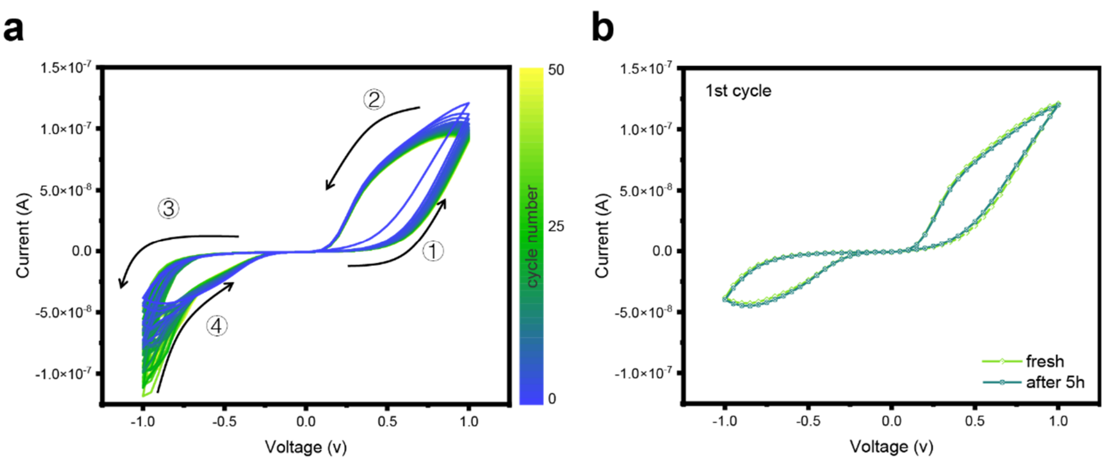

Supplementary Figure12. **Device IV characteristics under continuous scanning. a** *I*-*V* curves of fresh devices with multiple number of cycles. **b** Recovery after 5 h. (scanning direction: 0 V→ +1 V→ 0 V→ -1 V, scanning rate: 0.05 V·s^{-1})

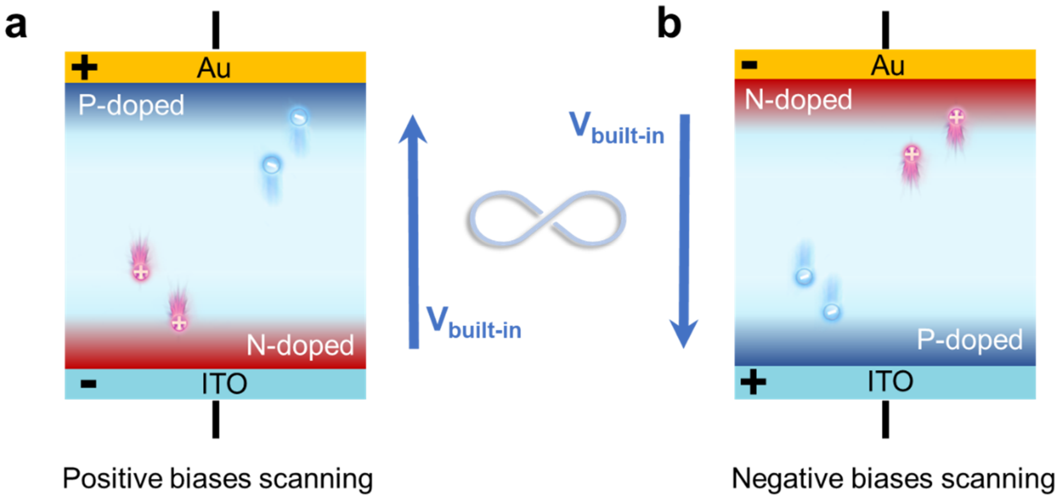

Supplementary Figure13. **Ion-drift mechanism of device under different polarity bias. a** During positive-biases. **b** During negative-bias scanning.

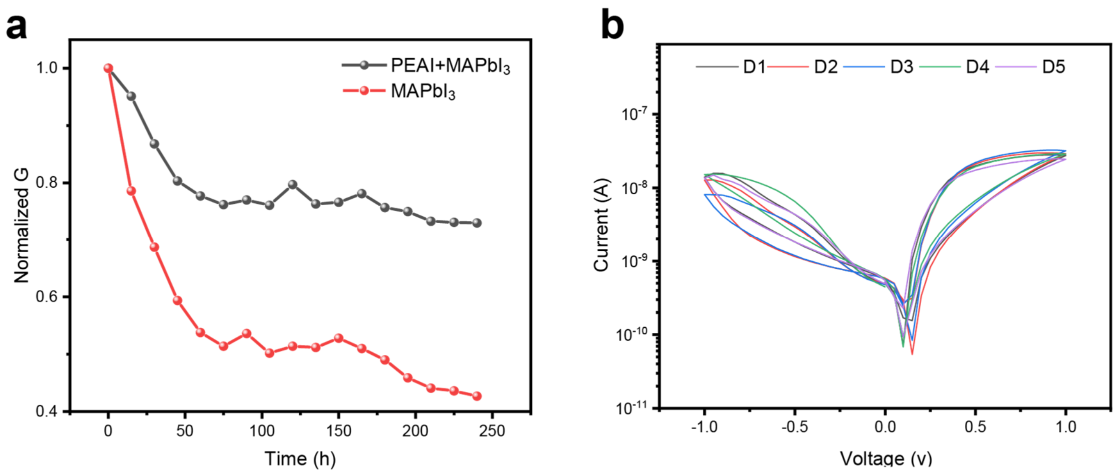

Supplementary Figure14. **Stability and uniformity of the device. a** Change in normalized device conductance vs duration of exposure to ambient air (RH 50% ~ 60%, RT, darkness). **b** 1st-cycle *I–V* characteristic of five PEAI-passivated devices after exposure to air for 240 h (scanning directions: 0 V→ +1 V→ 0 V→ -1 V, scanning rate: 0.05 V·s^{-1}).

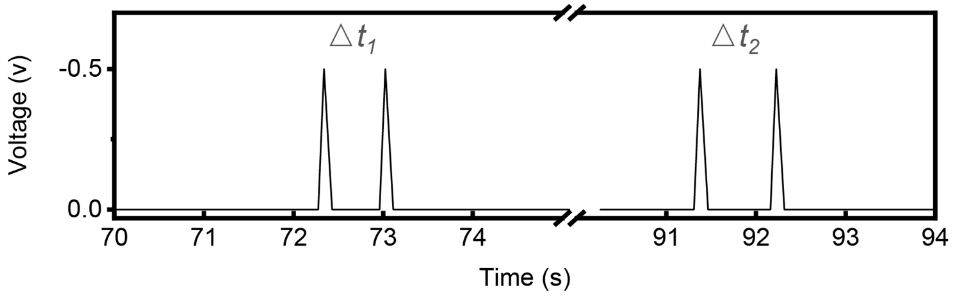

Supplementary Figure15. **Excitation signals of PPF**, a pair of spikes (-0.5 V, 68 ms) with Δt_1 = 680 ms and Δt_2 = 850 ms.

5. The current response under different voltages

In statistical analysis, the residual is the difference between the observed value and the

value estimated by curve-fitting, and is usually defined in connection with linear models[S38]. Here, to enable intuitive comparison of the change of linearity before and after passivation, we use the residual ratio

$$\text{Residual ratio}=\frac{\sqrt{(I_F-I_R)^2}}{I_R}, \tag{2}$$

where I_F represents the current fitting value under presynaptic voltage, and I_R represents the corresponding measured value.

We performed linear regression analysis on the data in the high-voltage area (Supplementary Fig.16a,b), and calculated the residual ratio (Supplementary Fig.16 c). It was much higher in the non-passivated synapses than in the passivated ones.

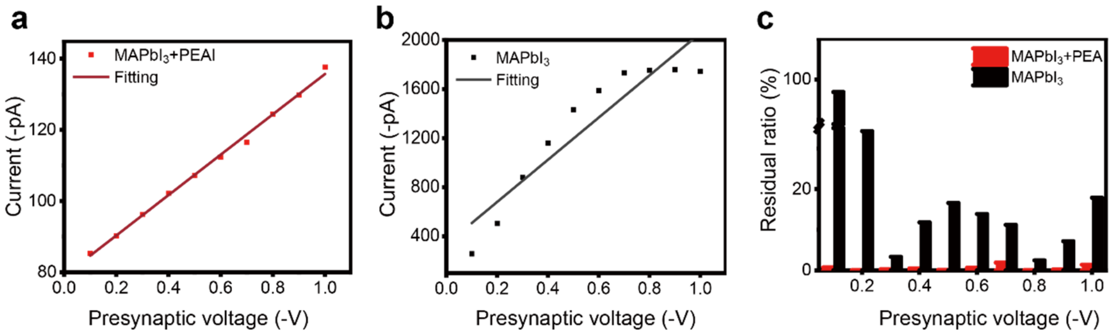

Supplementary Figure16. **Current response under different voltages. a** The value of EPSC and fitted line vs voltage in passivated perovskite artificial synapses. **b** The value of EPSC and fitted line vs voltage in perovskite artificial synapses without passivation. **c** Histogram of residual ratio comparison before and after passivation.

The synaptic current decreased significantly with the decrease of excitation voltage.The small current response of artificial synapses is attractive because it is beneficial for low energy consumption and the connection and functional coupling of a hybrid nervous system in the future. However, a small current is easily disturbed by noise, so the need to read a small current imposes very high requirements on the equipment.

6. Ion migration in RT-PVK artificial synapses

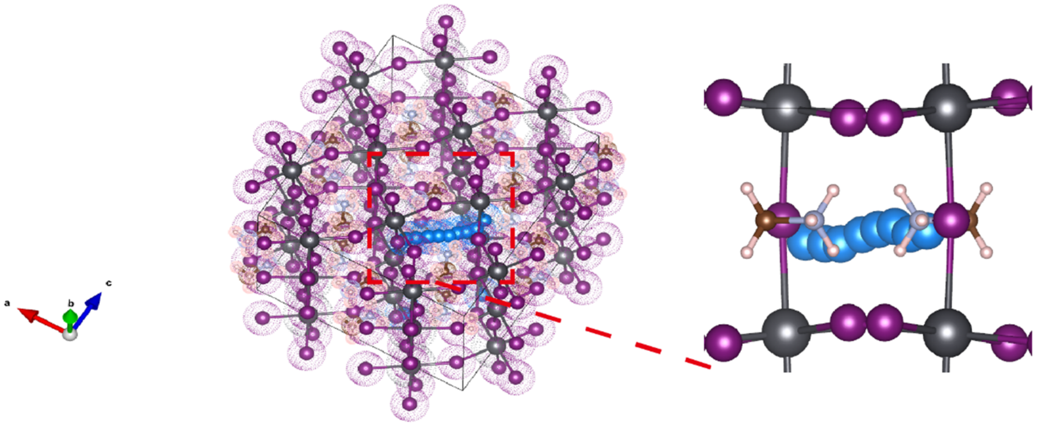

Supplementary Figure17.**Path 2 that lateral movement through the gap of MA^+.** The locations during the migration are shown in blue balls.

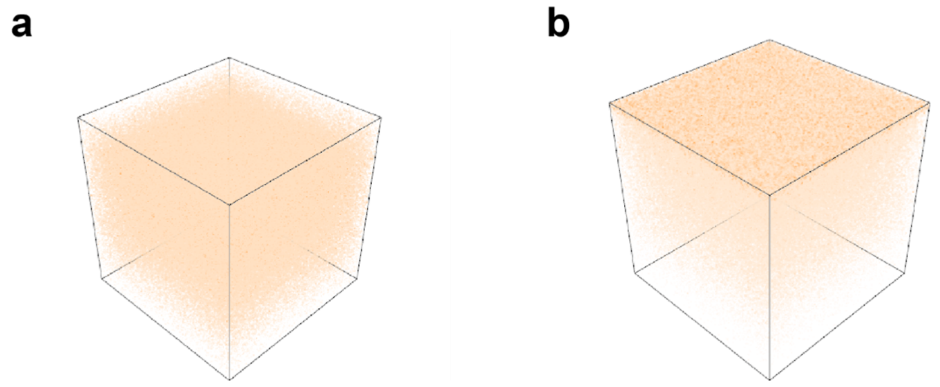

Supplementary Figure18. **3D tomography results of TOF-SIMS for distribution of I^-. a** Distribution before applying 500 spikes of 15 mV. **b** Distribution after applying 500 spikes of 15 mV.

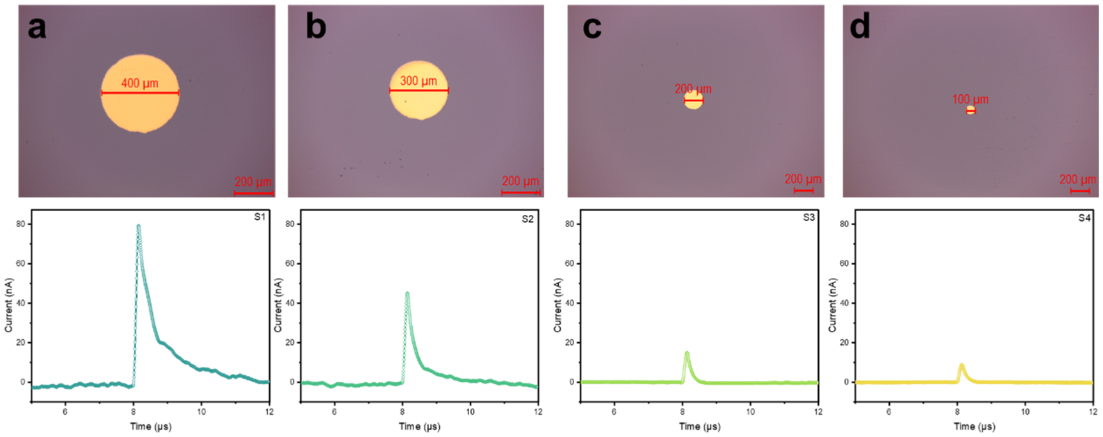

Supplementary Figure19. **Size information of devices. a** Optical photograph of device S1 with diameter 400 μm, and its EPSC under a single spike (15 mV, 100 ns). **b** Optical photograph of device S2 with diameter 300 μm, and its EPSC under a single spike (15 mV, 100 ns). **c** Optical photograph of device S3 with diameter 200 μm, and its EPSC under a single spike (15 mV, 100 ns). **d** Optical photograph of device S4 with diameter 100 μm, and its EPSC under a single spike (15 mV, 100 ns).

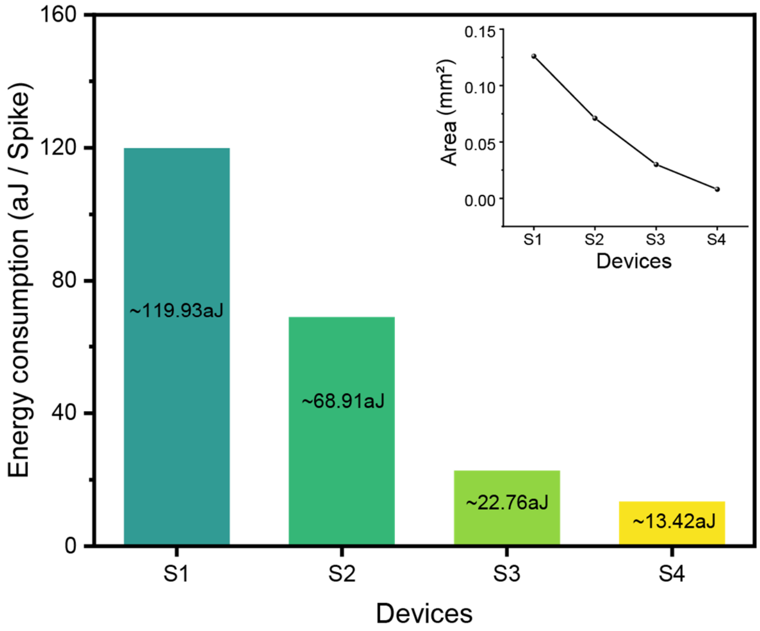

Supplementary Figure20. **Energy consumption statistics of devices with different sizes.** Inset: areas data of the devices.

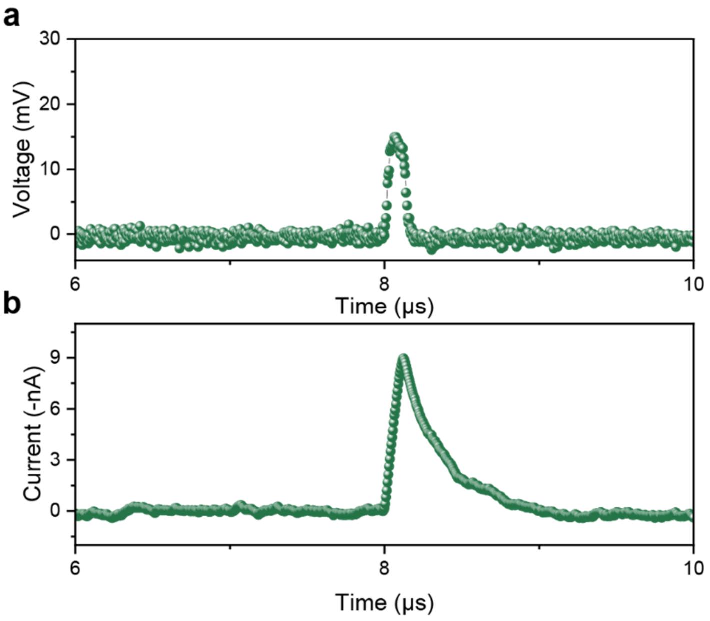

Supplementary Figure21. **ESPC peak of a PEAI-passivated device as triggered by a presynaptic spike (15 mV, 100 ns). a** 15mV excitation voltage signal with 100ns pulse width. **b** ESPC under a 15mV,100ns excitation voltage.

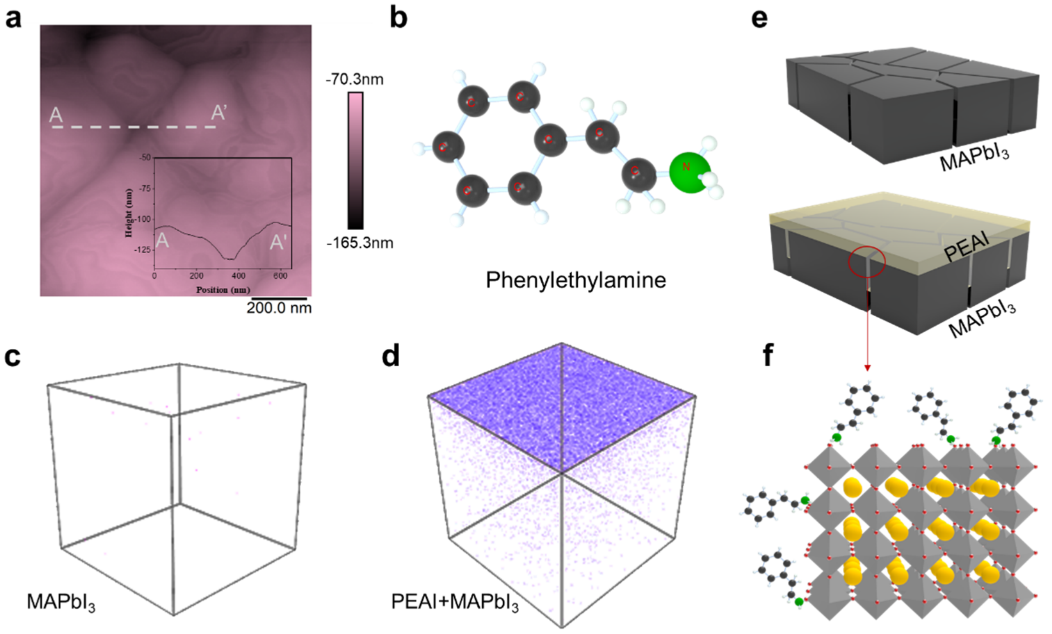

Supplementary Figure22. **Passivation of PEAI a** AFM height images at grain boundaries of RT-PVK (Inset: height contour distribution along A-A’). **b** Structure of

phenylethylamine. **c, d** 3D tomography results of TOF-SIMS for distribution of $C_6H_5^-$ in perovskite bulk before and after PEAI passivation. **e** Schematic illustration of passivation by PEAI.

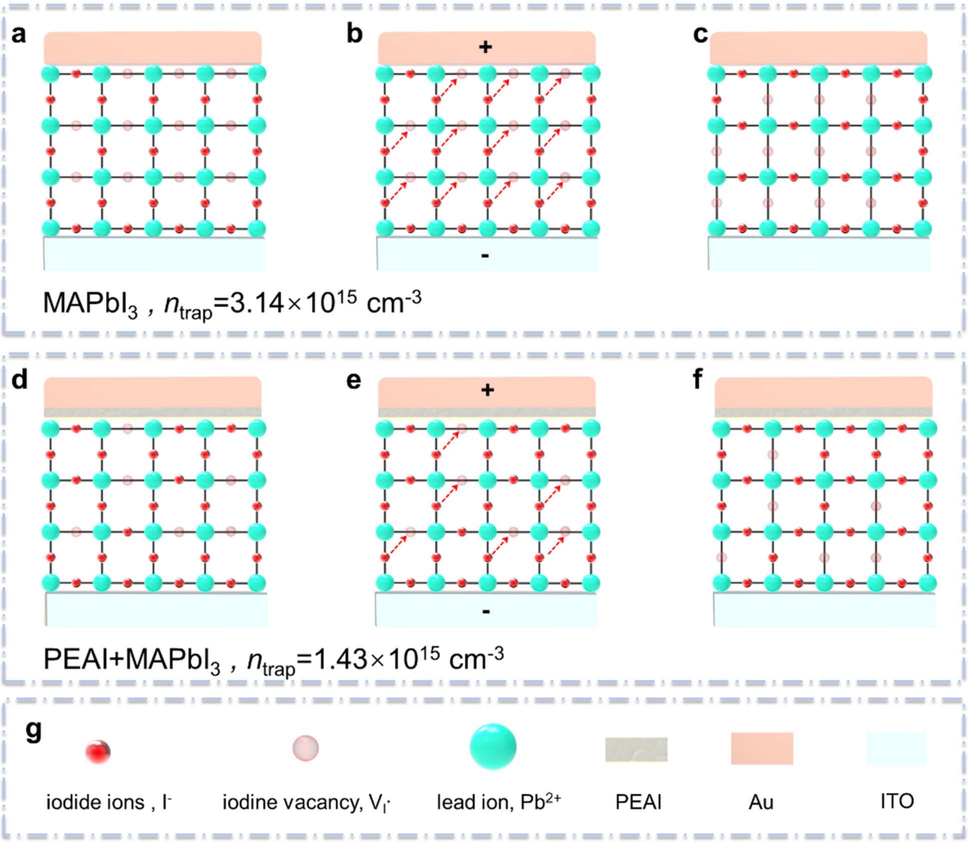

Supplementary Figure23. **Comparison of ion migration before and after passivation.** **a** $MAPbI_3$ crystal without passivation includes many defects, such as iodine vacancies. **b** Iodide ions hop to iodine vacancies when an external electric field is present. **c** After the ion migrates, its original position becomes a new vacancy. **d** Passivation of $MAPbI_3$ by PEAI reduces the number of defects, so **e** the number of iodide ions that hop to iodine vacancies under the applied electrical is reduced. **f** Fewer vacancies form.

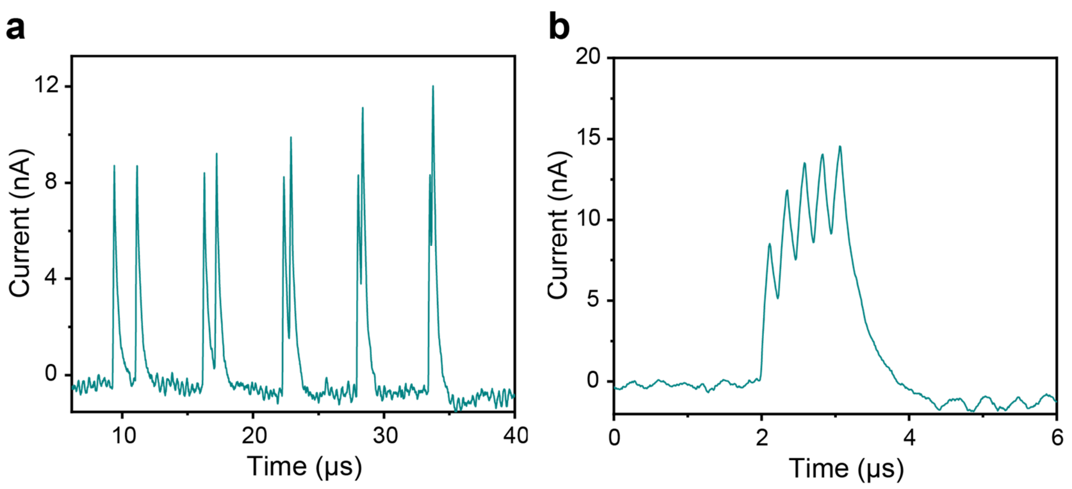

Supplementary Figure24. **Synaptic plasticity of ultrahigh-frequency mode. a** EPSC triggered by pairs of spikes (15 mV, 100 ns). **b** EPSC triggered by five consecutive spikes (15 mV, 100 ns).

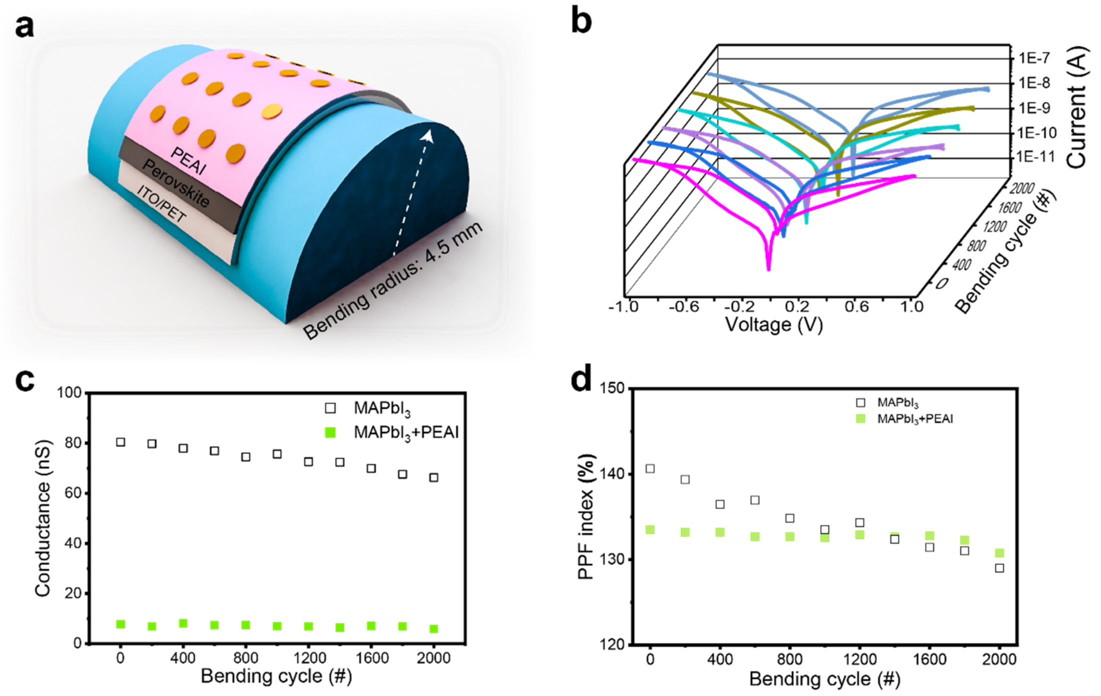

Supplementary Figure25 **Flexibility of the device. a** Configuration diagram of flexibility measurement in curve state. **b** *I-V* sweep curves at various bending cycles. **c** Variation of conductance in perovskite artificial synapse with/without PEAI passivation after repeated bending. **d** Change of PPF index in perovskite artificial synapse with/without PEAI passivation after bending.

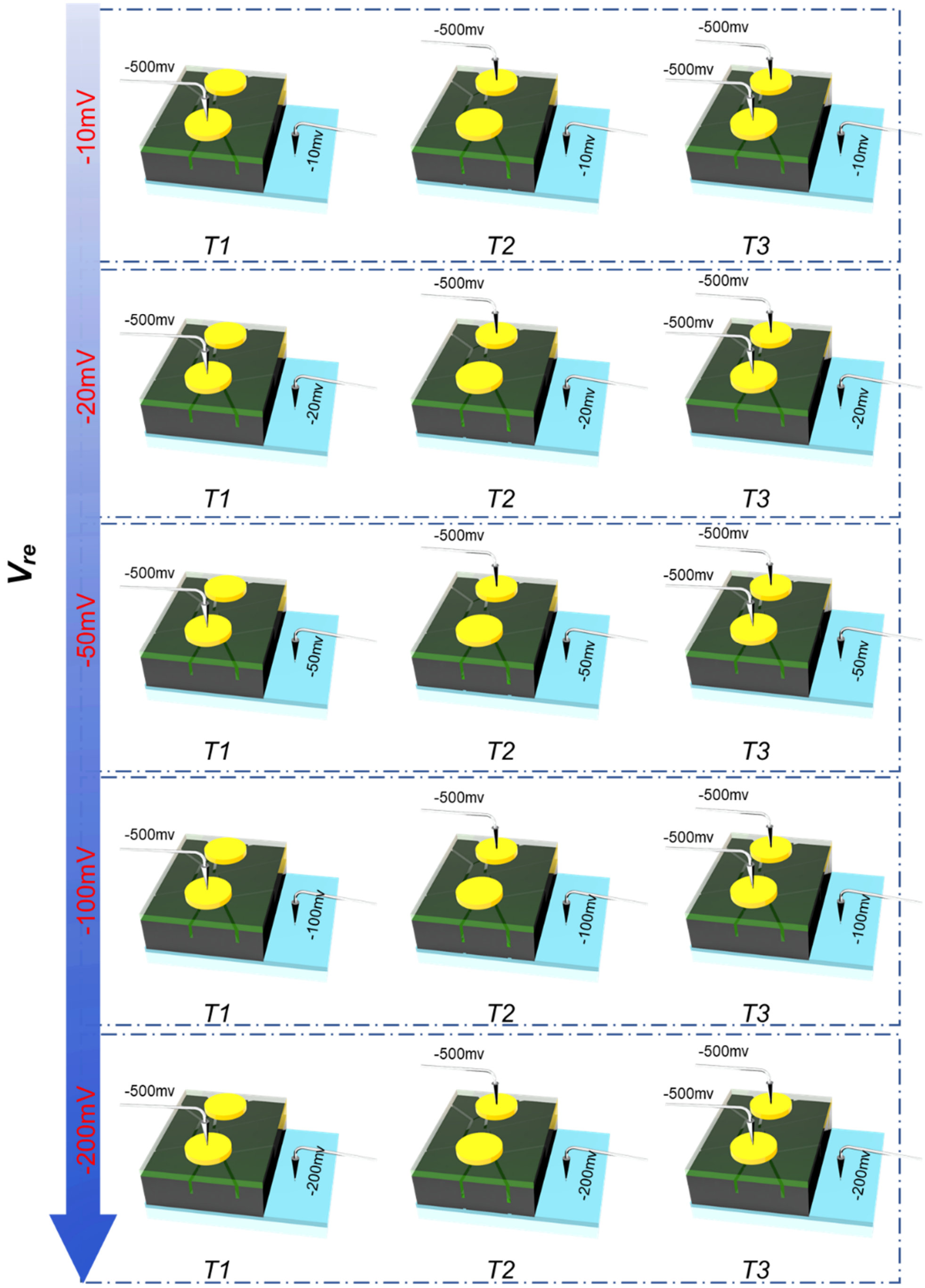

Supplementary Figure26. **Schematic diagram of logical operation function. a** Figure 4C of the original manuscript. **b** Changing the Vre applied to ITO to achieve logical operations.

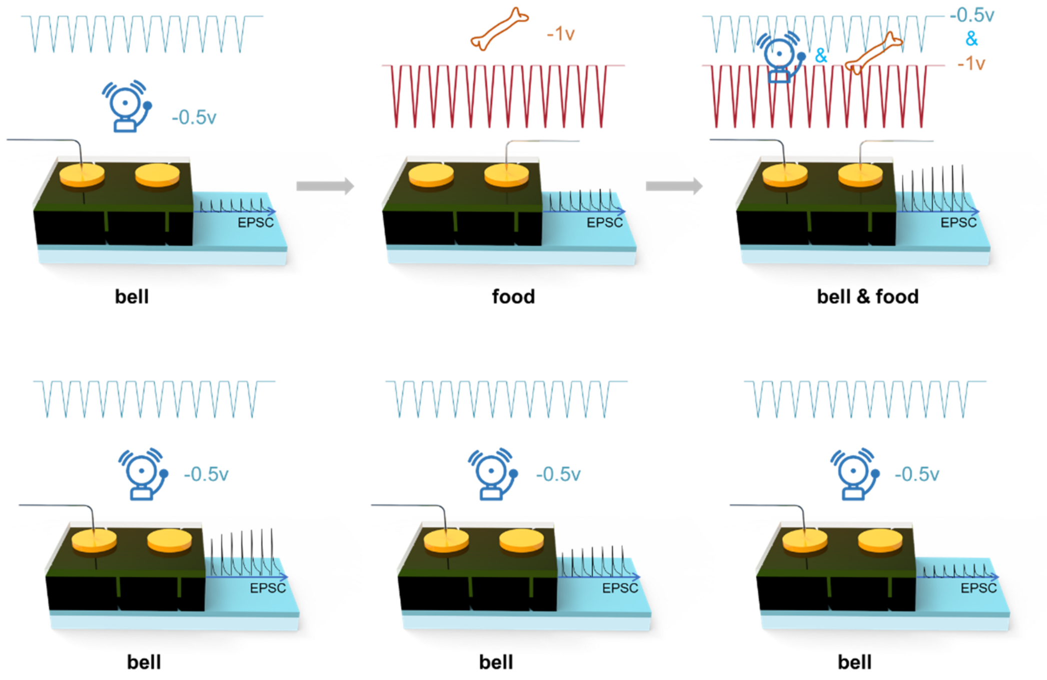

Supplementary Figure27. **Schematic diagram of Pavlovian learning.** Training process of artificial synapses to realize conditioning.

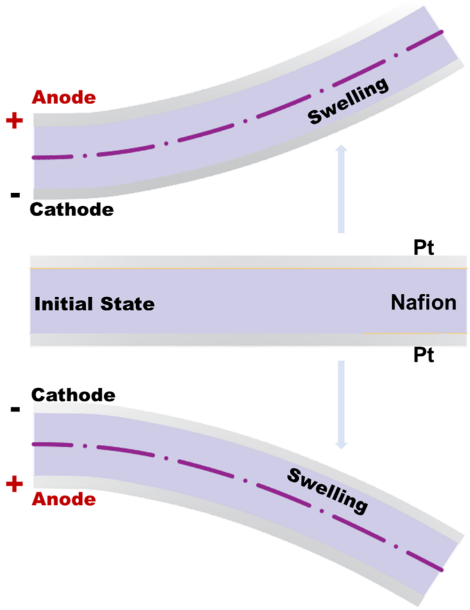

Supplementary Figure28. **Structure and motion mechanism of IPMC artificial muscle.** After hydrated cation migration, swelling occurs on the cathode side, causing IPMC to bend to the anode.

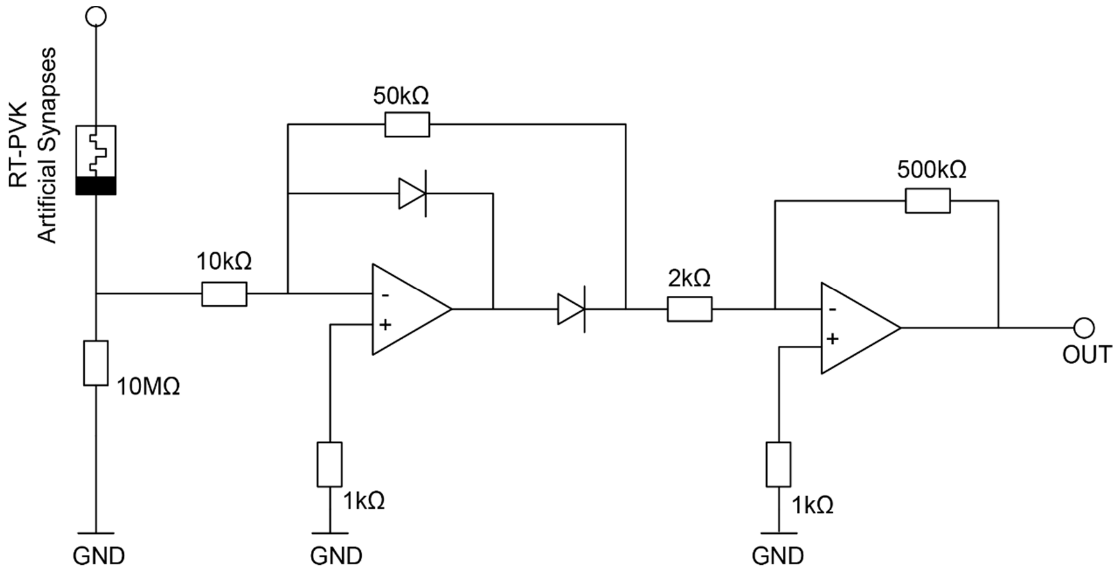

Supplementary Figure29. **Accessory circuits.** Current-to-voltage conversion and amplifying circuit.

Supplementary Table

Supplementary Table 1. Summary of Artificial synapse energy consumption

**Active Channel of synapse device**	**Structure**	**V_{read} (mV)**	**E_C (aj)**	**Ref.**
Biological synapses	\	\	~10^4	S1
α-In_2Se_3 Nanosheets	3T	2×10^3	3.36×10^3	S2
$CsPbI_2Br$ perovskite nanocrystals /IGZO	3T	\	2.6×10^6	S3
Single electrospun PQT-12/PEO fiber	3T	0.5	≈3.9×10^3	S4
P3HT/PEO	3T	1	~1.23×10^3	S5
InGaAs nanowires	3T	5×10^3	840	S6
In/MoS_2	3T	\	68.9	S7
DPP-DTT/$CsPbBr_3$ QDs	3T	0.5	500	S8
chlorophyll/OSC	3T	1×10^{-2}	25	S9
organic single-crystalline nanoribbon	3T	1	290	S10
MoS_2/Al_2O_3 /ZrO_2	3T	500	18.3	S11
MXene	2T	80	6.3×10^6	S12
single-crystal MoS_2	2T	5×10^3	1.8×10^6	S13
BCP/ $MAPbBr_3$ nanocrystals	2T	\	5.8×10^6	S14
N-doped ZnO	2T	4×10^3	5.6×10^4	S15
$CH_3NH_3PbBr_3$ single-crystal	2T	300	≈1.8×10^4	S16
Zeolite-Ag	2T	2×10^3	7.5×10^3	S17
PMTAA	2T	30	6.9×10^3	S18
P3HT-b-PBA	2T	0.3	560	S19
NaAc doped PVA	2T	1	132	S20
**PEAI/ RT- $MAPbI_3$**	**2T**	**15**	**≈13.5**	**※This work**

E_C: energy consumption; V_{read}: reading voltage

Supplementary Table 2. Summary of perovskite artificial synapse

Artificial synapse device	Type	Temp (°C)	RP Freq	RP time	Bending (times)	Radius	Ref.
Glass /ITO/ BCCP $(PEA)_2PbBr_4$ /Al	U	90	25Hz	\	\	\	S21
Glass /ITO/$FAPbBr_3$/Al	U	50	20Hz	\	\	\	S22
Au/Pentacence/PS/BCP/ $MAPbBr_3/SiO_2$	U	70	50 Hz	\	\	\	S14
Au/ $(R\text{-}MPEA)_{1.5}PbBr_{3.5}(DMSO)_{0.5}/SiO_2$	U	50	20Hz	\	\	\	S23
Au/P(VDF-TrFE)/ CsPbBr3/ Au /Glass	U	70	1KHz	\	\	\	S24
Au/ (MAPbBr3) single-crystalline/ Au /Glass	U	120	20 Hz	\	\	\	S16
Au/ $MA_3Sb_2Cl_9$ /ITO /Glass	U	100	2 Hz	\	\	\	S25
Glass /ITO/ $(3AMP)PbI_4$/Al	U	100	\	\	\	\	S26
Cr/Au/ Si-NC/perovskite/ SiO_2	U	100	4 Hz	\	\	\	S27
PMMA/ Au/ $MAPbI_3$/ Si-NC/ SiO_2	U	100	4.35Hz	\	\	\	S28
Ag/$FA_{0.9}Cs_{0.1}PbI_{3-x}Cl_x$/spiro-MeOTAD /ITO /Glass	U	\	\	\	\	\	S29
Au/ $CsPbBr_3$PNs/CuSCN/PEDOT:PSS/ITO	U	150	2 Hz	\	\	\	S30
Ag/PMMA/ $Cs_2AgBiBr_6$ /ITO /Glass	U	60	3KHz	\	\	\	S31
PET/$SnO_2/CsPbCl_3$/TAPC/TAPC:MoO_3/MoO_3/Ag /MoO_3	F	120	10 Hz	0.5s	\	\	S32
$(PEA)_2SnI_4$/ rGO/ PEDOT:PSS/ PET	F	90	20 Hz	0.63s	1500	5.5 mm	S33
Au/$CsPbBr_3$QDsDPP-DTT/ Au	F	60	20 Hz	0.5s	1000	5 mm	S34
Au/BPE-PTCDI /$CH_3NH_3PbBr_3/P_2VP$/PEN	F	140	\	\	1000	5 mm	S35
Ag/$CsSnCl_3$ /ITO /Glass	F	\	\	10μs	200	12 mm	S36
Pt/$BiFeO_3$/LSMO/PDMS	F	800	\	500ns	100	8mm	S37
**Au/PEAI/ RT-$MAPbI_3$/ITO/ PET**	**F**	**R.T.**	**4.17MHz**	**100ns**	**2000**	**4.5 mm**	**※This work**

F: device is flexible; U: device is inflexible. Temp: preparation temperature of the device. RP Freq: maximum test frequency of the device. RP time: minimum response pulse width that can still be achieved after the device is bent.

Supplementary Reference

S1 Gao, J. *et al.* Intrinsic polarization coupling in 2D α-In_2Se_3 toward artificial synapse with multimode operations. *SmartMat* **2**, 88-98 (2021).

S2 Tang, B., Li, X., Liao, J. & Chen, Q. Ultralow power consumption and large dynamic range synaptic transistor based on α-In_2Se_3 nanosheets. *ACS Appl. Electron. Mater.* **4**, 598-605 (2022).

S3 Cao, Y. *et al.* Ultralow light-power consuming photonic synapses based on ultrasensitive perovskite / indium-gallium-zinc-oxide heterojunction phototransistors. *Adv. Electron. Mater.* **8**, 2100902 (2022).

S4 Liu, D., Shi, Q., Dai, S. & Huang, J. The design of 3D-interface architecture in an ultralow-power, electrospun single-fiber synaptic transistor for neuromorphic computing. *Small* **16**, 1907472 (2020).

S5 Xu, W., Min, S.-Y., Hwang, H. & Lee, T.-W. Organic core-sheath nanowire artificial synapses with femtojoule energy consumption. *Sci. Adv.* **2**, e1501326 (2016).

S6 Xie, P. *et al.* Ferroelectric p(vdf-trfe) wrapped ingaas nanowires for ultralow-power artificial synapses. *Nano Energy* **91**, 106654 (2022).

S7 Hu, Y. *et al.* Ultralow power optical synapses based on MoS_2 layers by indium-induced surface charge doping for biomimetic eyes. *Adv. Mater.* **33**, 2104960 (2021).

S8 Hao, D., Zhang, J., Dai, S., Zhang, J. & Huang, J. Perovskite/organic semiconductor-based photonic synaptic transistor for artificial visual system. *ACS Appl. Mater. Interfaces* **12**, 39487-39495 (2020).

S9 Yang, B. *et al.* Bioinspired multifunctional organic transistors based on natural chlorophyll/organic semiconductors. *Adv. Mater.* **32**, 2001227 (2020).

S10 Zhang, C. *et al.* Sub-femtojoule-energy-consumption conformable synaptic transistors based on organic single-crystalline nanoribbons. *Adv. Funct. Mater.* **31**, 2007894 (2021).

S11 Wang, T.-Y. *et al.* Ultralow power wearable heterosynapse with photoelectric

synergistic modulation. *Adv. Sci.* **7**, 1903480 (2020).

S12 Wei, H. *et al.* Redox mxene artificial synapse with bidirectional plasticity and hypersensitive responsibility. *Adv. Funct. Mater.* **31**, 2007232 (2021).

S13 Shen, J. *et al.* Low consumption two-terminal artificial synapse based on transfer-free single-crystal MoS_2 memristor. *Nanotechnology* **31**, 265202 (2020).

S14 Lee, K. *et al.* Retina-inspired structurally tunable synaptic perovskite nanocones. *Adv. Funct. Mater.* **31**, 2105596 (2021).

S15 Lin, Y. *et al.* Nitrogen-induced ultralow power switching in flexible zno-based memristor for artificial synaptic learning. *Appl. Phys. Lett.* **118**, 103502 (2021).

S16 Gong, J. *et al.* Lateral artificial synapses on hybrid perovskite platelets with modulated neuroplasticity. *Adv. Funct. Mater.* **30**, 2005413 (2020).

S17 Zeng, T. *et al.* Zeolite-based memristive synapse with ultralow sub-10-fj energy consumption for neuromorphic computation. *Small* **17**, 2006662 (2021).

S18 Jiang, C. *et al.* Efficient two-terminal artificial synapse based on a network of functionalized conducting polymer nanowires. *J. Mater. Chem. C* **7**, 9933-9938 (2019).

S19 Yang, W.-C. *et al.* Low-energy-consumption and electret-free photosynaptic transistor utilizing poly(3-hexylthiophene)-based conjugated block copolymers. *Adv. Sci.* **9**, 2105190 (2022).

S20 Hu, L. *et al.* Ultrasensitive freestanding and mechanically durable artificial synapse with attojoule power based on na-salt doped polymer for biocompatible neuromorphic interface. *Adv. Funct. Mater.* **31**, 2106015 (2021).

S21 Kim, S.-I. *et al.* Dimensionality dependent plasticity in halide perovskite artificial synapses for neuromorphic computing. *Adv. Electron. Mater.* **5**, 1900008 (2019).

S22 Das, U., Sarkar, P., Paul, B. & Roy, A. Halide perovskite two-terminal analog memristor capable of photo-activated synaptic weight modulation for neuromorphic computing. *Appl. Phys. Lett.* **118**, 182103 (2021).

S23 Gong, J. *et al.* An air-stable two-dimensional perovskite artificial synapse. *Semicond. Sci. Technol.* **35**, 104001 (2020).
S24 Jeong, B., Gkoupidenis, P. & Asadi, K. Solution-processed perovskite field-effect transistor artificial synapses. *Adv. Mater.* **33**, 2104034 (2021).
S25 Ni, Z. *et al.* E-synapse based on lead-free organic halide perovskite $(CH_3NH_3)_3Sb_2Cl_9$ for neuromorphic computing. *IEEE Trans. Electron Devices* **68**, 4425-4430 (2021).
S26 Park, Y. & Lee, J.-S. Controlling the grain size of dion–jacobson-phase two-dimensional layered perovskite for memory application. *ACS Appl. Mater. Interfaces* **14**, 4371-4377 (2022).
S27 Zhu, Y. *et al.* Perovskite-enhanced silicon-nanocrystal optoelectronic synaptic devices for the simulation of biased and correlated random-walk learning. *Research* **2020**, 7538450 (2020).
S28 Yin, L. *et al.* Optically stimulated synaptic devices based on the hybrid structure of silicon nanomembrane and perovskite. *Nano Lett.* **20**, 3378-3387 (2020).
S29 Huang, L. *et al.* All in one: A versatile n-perovskite/p-spiro-meotad p–n heterojunction diode as a photovoltaic cell, photodetector, and memristive photosynapse. *J. Phys. Chem. Lett.* **12**, 12098-12106 (2021).
S30 Ma, F. *et al.* Optoelectronic perovskite synapses for neuromorphic computing. *Adv. Funct. Mater.* **30**, 1908901 (2020).
S31 Lao, J. *et al.* An air-stable artificial synapse based on a lead-free double perovskite $Cs_2AgBiBr_6$ film for neuromorphic computing. *J. Mater. Chem. C* **9**, 5706-5712 (2021).
S32 Yang, L. *et al.* Transparent and flexible inorganic perovskite photonic artificial synapses with dual-mode operation. *Adv. Funct. Mater.* **31**, 2008259 (2021).
S33 Qian, L. *et al.* A lead-free two-dimensional perovskite for a high-performance flexible photoconductor and a light-stimulated synaptic device. *Nanoscale* **10**, 6837-6843 (2018).
S34 Zhang, J. *et al.* Tailoring neuroplasticity in flexible perovskite QDs-based

optoelectronic synaptic transistors by dual modes modulation. *Nano Energy* **95**, 106987 (2022).

S35 Yang, W.-C. *et al.* Comprehensive non-volatile photo-programming transistor memory via a dual-functional perovskite-based floating gate. *ACS Appl. Mater. Interfaces* **13**, 20417-20426 (2021).

S36 Siddik, A. *et al.* Nonvolatile resistive switching and synaptic characteristics of lead-free all-inorganic perovskite-based flexible memristive devices for neuromorphic systems. *Nanoscale* **13**, 8864-8874 (2021).

S37 Zhao, Z. *et al.* Flexible artificial synapse based on single-crystalline $BiFeO_3$ thin film. *Nano Research* **15**, 2682-2688 (2022).

S38 Cox DR, Snell EJ. A General Definition of Residuals. *J. R. Stat. Soc. B* **30**, 248-265 (1968).

S39 Kresse G, Furthmüller J. Efficient iterative schemes for ab initio total-energy calculations using a plane-wave basis set. *Phys. Rev. B* **54**, 11169-11186 (1996).

S40 Perdew JP, Burke K, Ernzerhof M. Generalized Gradient Approximation Made Simple. *Phys. Rev. Lett.* **77**, 3865-3868 (1996).

S41 Kresse G, Joubert D. From ultrasoft pseudopotentials to the projector augmented-wave method. *Phys. Rev. B* **59**, 1758-1775 (1999).

S42 Blöchl PE. Projector augmented-wave method. *Phys. Rev. B* **50**, 17953-17979 (1994).

S43 Grimme S, Antony J, Ehrlich S, Krieg H. A consistent and accurate ab initio parametrization of density functional dispersion correction (DFT-D) for the 94 elements H-Pu. *J. Chem. Phys.* **132**, 154104 (2010).

S44 Henkelman G, Uberuaga BP, Jónsson H. A climbing image nudged elastic band method for finding saddle points and minimum energy paths. *J. Chem. Phys.* **113**, 9901-9904 (2000).
