# Supplementary material for: A bioinspired flexible neuromuscular system based thermal-annealing-free perovskite with passivation
Source: Nat Commun. 2022 Dec 2;13:7427. doi: 10.1038/s41467-022-35092-w (PMC9718817; doi:10.1038/s41467-022-35092-w)
Supplement: Supplementary file 2 — Description of Additional Supplementary Files [file 41467_2022_35092_MOESM2_ESM.docx]

**Description of Additional Supplementary Files**

File Name: Supplementary Movie 1

Description: The formation process of perovskite at room temperature.

File Name: Supplementary Movie 2

Description: EPSC and ion migration in low frequency mode.
